# Blockade of Na/H exchanger stimulates glioma tumor immunogenicity and enhances combinatorial TMZ and anti-PD-1 therapy

**DOI:** 10.1038/s41419-018-1062-3

**Published:** 2018-09-27

**Authors:** Xiudong Guan, Md Nabiul Hasan, Gulnaz Begum, Gary Kohanbash, Karen E. Carney, Victoria M. Pigott, Anders I. Persson, Maria G. Castro, Wang Jia, Dandan Sun

**Affiliations:** 1 Department of Neurosurgery, Beijing Tiantan Hospital, Capital Medical University, Beijing, China; 2 Department of Neurology, University of Pittsburgh, Pittsburgh, PA USA; 3 Chinese National Clinical Research Center for Neurological Diseases, Beijing, China; 4 Department of Neurological Surgery, University of Pittsburgh, Pittsburgh, PA USA; 5 Department of Neurology, University of California, San Francisco, CA USA; 6 Department of Neurological Surgery, University of California, San Francisco, CA USA; 7 Department of Neurological Surgery, University of Michigan Medical School, Ann Arbor, MI USA; 8 Beijing Neurosurgical Institute, Beijing, China

## Abstract

The weak immunogenicity of gliomas presents a barrier for effective immunotherapy. Na/H exchanger isoform 1 (NHE1) maintains alkaline intracellular pH (pH_i_) of glioma cells and acidic microenvironment. In addition, NHE1 is expressed in tumor-associated microglia and tumor-associated macrophages (TAMs) and involved in protumoral communications between glioma and TAMs. Therefore, we hypothesize that NHE1 plays a role in developing tumor resistance and immunosuppressive tumor microenvironment. In this study, we investigated the efficacy of pharmacological inhibition of NHE1 on combinatorial therapies. Here we show that temozolomide (TMZ) treatment stimulates NHE1 protein expression in two intracranial syngeneic mouse glioma models (SB28, GL26). Pharmacological inhibition of NHE1 potentiated the cytotoxic effects of TMZ, leading to reduced tumor growth and increased median survival of mice. Blockade of NHE1 stimulated proinflammatory activation of TAM and increased cytotoxic T cell infiltration into tumors. Combining TMZ, anti-PD-1 antibody treatment with NHE1 blockade significantly prolonged the median survival in the mouse glioma model. These results demonstrate that pharmacological inhibition of NHE1 protein presents a new strategy for potentiating TMZ-induced cytotoxicity and increasing tumor immunogenicity for immunotherapy to improve glioma therapy.

## Introduction

Patients with glioblastoma (GBM), World Health Organization grade IV tumor, presently persist short median post-diagnosis survival time (approximately 20 months), despite of surgical resection followed by radiotherapy and temozolomide (TMZ)-mediated chemotherapy^1–3^. The alkylating agent TMZ produces lethal DNA lesions and is the first-line chemotherapeutic agent for glioma. However, the obstacles in glioma therapy include acquired resistance to TMZ-mediated DNA damage via the function of DNA repair protein O6-methylguanine-DNA methyltransferase (MGMT), incomplete surgical resection due to the highly aggressive behavior of glioma and glioma stem cells, and tumor-supportive microenvironment^4–6^. Newly developed cancer immunotherapy provides promising survival benefits in some patients^7^, but other patients are not responsive to the therapy^8–11^ and tumor relapse is common^12,13^. Successful antitumor immunotherapy depends on an immunogenic tumor microenvironment and the interactions between cancer cells and enhanced T cell antitumor immunity^14^. However, a non-immunogenic, immunosuppressive tumor microenvironment may lead to exiguous clinical benefit^15^.

Na/H exchanger isoform 1 (NHE1) plays an important role in the progression of GBM^16^. NHE1 (*SLC9A1*) mRNA expression in GBM tumor tissues correlates with worse patient outcome in the Repository for Molecular Brain Neoplasia Data (REMBRANDT)^17^. We and other groups have demonstrated that NHE1 drives H^+^ efflux in exchange of Na^+^ influx to maintain an intracellular pH (pH_i_) of 7.3–7.5 in glioma cells^17,18^, a driving force for glycolytic metabolism^19^. Cancer cells rely on oxidative glycolysis by increasing glucose uptake and lactate production even in the presence of oxygen and fully functioning mitochondria, a process known as Warburg effect^20,21^. We recently showed that pharmacological inhibition of NHE1 inhibited proliferation and invasiveness in patient-derived GBM cell cultures and glioma-mediated activation of tumor associated microglia/macrophages^22^. TMZ treatment increased NHE1 protein levels in patient-derived GBM cells as a novel mechanism of TMZ resistance^17^. However, it remains unknown whether pharmacological inhibition of NHE1 potentiates TMZ-induced cytotoxicity and modifies the immunosuppressive tumor microenvironment to enhance the antitumor immunity in preclinical GBM animal models.

In this study, we report a positive correlation between elevated NHE1 mRNA expression (*SLC9A1*) and shorter overall survival of glioma patients in the GSE16011 dataset. In addition, NHE1 mRNA expression was higher in matched recurrent glioma compared with primary glioma in The Cancer Genome Atlas (TCGA) dataset. In the preclinical GBM mouse models, the combination therapy of TMZ with the NHE1 inhibitor HOE642 potentiated the cytotoxic effects of TMZ. Combining TMZ and anti-PD-1 antibody therapies with NHE1 blockade significantly prolonged the median survival in the mouse glioma model. Thus our results demonstrate that elevated NHE1 expression in glioma and tumor-associated macrophages (TAMs) are important for GBM progression and inhibition of NHE1 function represents a novel strategy to improve the efficacy of glioma therapies.

## Materials and methods

### Data collection

RNA microarray data was obtained from GSE16011 (https://www.ncbi.nlm.nih.gov/geo/query/acc.cgi?acc=GSE16011, *n* = 271). The overall survival was calculated from the date at the initial diagnosis to the date of death or last follow-up. Patients in the cohort were divided into two groups (high expression and low expression) according to the median SLC9A1 mRNA expression. The RNA sequencing data of 20 paired patients (primary glioma and matched recurrent glioma) were from TCGA (https://portal.gdc.cancer.gov/). The logarithmic transformation was used for data analysis.

### Cell cultures and authentication

The glioma GL26-mCitrine cells (GL26-cit) in adherent cultures, obtained from Professor Maria Castro, PhD, were derived as described previously^23^ and maintained in Dulbecco’s modified Eagle’s medium/4-(2-hydroxyethyl)-1-piperazineethanesulfonic acid (DMEM/HEPES) supplemented with 10% heat-inactivated fetal bovine serum (FBS), 2 mM L-glutamine, 1× Penicillin/streptomycin, and 600 µg/mL G418 sulfate (for selection of the mCitrine expression vector). The mouse glioma SB28-GFP cells, obtained from Professor Gary Kohanbash, PhD, were derived as described previously^24^ and seeded in DMEM/HEPES containing 10% heat-inactivated FBS, 2 mM L-glutamine, 1× Penicillin/streptomycin, and 1 mM sodium pyruvate. Cultures were passaged approximately every 4 days with fresh medium at a density of 10^6^ cells/75 cm^2^ in a culture flask (5–25 passages used in the study). All cell lines were authenticated by short tandem repeat DNA fingerprinting (by IDEXX BioResearch, Columbia, MO) in the past 6 months. In addition, PCR analysis was performed to confirm the absence of mycoplasma infection in all cell cultures.

### Immunoblotting assay

Cultured cells were washed with ice-cold phosphate-buffered saline (PBS) containing phosphoSTOP and protease inhibitors as described before^25^. Cells were lysed by sonication at 4 °C followed by protein quantification by measuring absorbance at 562 nm wavelength (BCA Protein Assay Kit). Samples and prestained molecular mass markers were denatured in sodium dodecyl sulfate (SDS) reducing buffer (1:2 vol/vol) and heated at 95 °C for 5 min. The samples were then electrophoretically separated on 4–15% SDS gels. After transferring to polyvinylidene difluoride membranes, the blots were blocked in 7.5% nonfat dry milk in Tris buffered saline for 1 h at room temperature (RT) and then incubated with a primary antibody at 4 °C overnight. Primary antibodies included mouse antibody against NHE1 (1:500), and β-actin antibody. After rinsing, the blots were incubated with horseradish peroxidase-conjugated secondary IgG (1:2000) for 1 h at RT. Bound antibody was visualized with an enhanced chemiluminescence assay. Protein band signal intensities were analyzed using ImageJ and normalized to β-actin expression. Full-size blot scans are available in supplementary materials.

### Syngeneic mouse glioma models

All animal experiments were approved by the University of Pittsburgh Institutional Animal Care and Use Committee and performed in accordance with the National Institutes of Health Guide for the Care and Use of Laboratory Animals.

C57BL/6 mice (female, 7–8 weeks old) were anesthetized with 2% isoflurane. Animals under anesthesia were placed into a stereotactic frame and a single midline incision was made to expose the cranium. A hole was drilled into the cranium above the left cerebral hemisphere using a precision power drill equipped with a fine bit at the following coordinates from bregma: +0.5 mm AP, +2.1 mm ML, and −3.2 mm DV. Using aseptic technique, upon exposing the underlying dura, 4 × 10^4^ cells (GL26-cit) or 5 × 10^4^ cells (SB28-GFP) in 2 μL of serum-free DMEM was injected into the right striatum using a micro-pump injector and a 5-μL Hamilton syringe equipped with a 33-gauge needle for 4 min at a rate of 500 nL/min. Cells were allowed to settle for 5 min followed by slow needle withdrawal. Ketofen (2 mg/kg, i.p.) was administrated once prior to surgery and daily for 2 days after the surgery. Animals were then allowed to recover in their cages under a heat lamp and access to water and wet chow.

### Drug treatment regimens

Starting 2 days after tumor cell implantation (d.p.i.), mice were randomly assigned to each treatment group and received the therapy for 5 consecutive days: vehicle control (1.25% dimethyl sulfoxide (DMSO) in PBS, 10 mL/kg/day, i.p.), NHE1 inhibitor HOE642 (H, 0.25 mg/kg, twice a day, i.p.), TMZ therapy (T, 2.5 mg/kg/day, i.p.), or T+H (T of 2.5 mg/kg/day+H of 0.25 mg/kg, twice a day, i.p.) combination treatment. For the immunotherapy, mice were treated with isotype IgG2a (10 mg/kg/day, i.p.), anti-PD-1antibody (10 mg/kg/day, i.p.), or T+H followed up with anti-PD-1 at 8, 10, and 12 d.p.i.

### Animal survival test

Tumor-bearing animals were monitored daily for signs of pain, discomfort, or neurological impairment. Signs of chronic pain, such as hunched posture, weight loss, absence of grooming behavior, and of neurological impairment, like seizures, weakness, difficulty walking, an inability to right themselves, circling behavior, and unusual aggressiveness or timidity were used to infer tumor development. In tumor cell-injected mice, a loss of 20% body weight, severe neurological impairment, or major loss in body scoring index (<2.0 on a 5-point scale) were used as the humane end point. All other surviving mice were sacrificed at 90 days after glioma cell injection.

### Evaluation of glioma tumor

At the termination point, animals were anesthetized with 3% isoflurane in 70% N_2_O and 30% O_2_ and exhibited no toe and tail reflexes. Animals were transcardially perfused with 0.9% saline solution using a 40 mL syringe followed by a solution of 4% paraformaldehyde (PFA) in PBS (pH7.4). Brains were harvested and stored in 4% PFA at 4 °C overnight, then stored in 30% sucrose for cryoprotection. Coronal tissue sections (25-μm thick) were made using a vibratome (Leica SM 2010R, Buffalo Grove, IL). To measure xenograft tumor size, the mCitrine-positive or green fluorescent protein (GFP)-positive tumor area in each brain section (bregma: +1.5, +1.0, +0.5, 0.0, −0.5, −1.0, −1.5, −2.0 mm AP) were selected and measured using the ImageJ software. Tumor volume was calculated (multiplying the sum of tumor area measurement by the height including section thickness and the *z* gap between slices)^26^. To define tumor core and border areas, under the ×40 oil immersion objective lens of Leica confocal microscope at 488 nm laser, the mCitrine-positive GL26 or GFP-positive SB28 tumor mass was identified. The center of the tumor mass with tightly packed mCitrine or GFP-positive glioma cells was defined as the tumor core, as described previously^27^. The tumor border (indicated as white dotted lines in Fig. 7) was defined as the area where mCitrine-positive or GFP-positive glioma cells were separated from the surrounding normal brain cells that do not contain either mCitrine or GFP signals.

### Proximity ligation assay (PLA) in cultured glioma cells and in glioma tumor tissues

PLA detects individual pairs of protein–protein interactions residing within 40 nm distance with a fluorophore-conjugated DNA probe in a polymerase-mediated rolling circle DNA amplification^28^. PLA assays were performed per the manufacturer’s instruction. Briefly, cultured GL26-Cit cells grown on glass coverslips or brain tissue sections (25 µm) from glioma-bearing animals were fixed in 4% PFA and incubated with mouse anti-NHE1, rabbit anti-MMP2, rabbit anti-MMP9, or rabbit anti-MT1-MMP primary antibodies (1:100) overnight at 4 °C. After washing unbound antibodies, proximity probes (anti-rabbit PLUS and anti-mouse MINUS) were applied and DNA was amplified after hybridization and ligation of oligonucleotide. PLA fluorescence of cultured glioma cells were captured by Nikon A-1 confocal microscope (×60). The fluorescence images of the brain tumor tissue sections were captured with a Leica DMIRE2 inverted confocal laser-scanning microscope under the ×40 oil immersion objective lens, with excitation at 488, 546, and 630 nm, and the emission fluorescence was recorded at 490–525, 556–573, and 650–750 nm, respectively.

### Immunofluorescence staining

Brain sections fixed in 4% PFA were mounted on microscope slides. Sections were then incubated with a blocking solution (0.3% Triton X-100/3% goat serum) for 60 min at RT and probed with primary antibodies (rabbit antibody against NHE1, 1:200, rabbit antibody against Ki67, 1:200, rabbit antibody against cleaved caspase-3, 1:200, or rat antibody against CD 8, 1:100) overnight at 4 °C. After rinsing in PBS 3 times for 15 min, tissue sections were incubated with respective secondary antibodies conjugated to Alexa Fluor® 546 (1:200 dilution) for 1 h at RT. Sections were then rinsed and incubated with To-pro-3 iodide (1:1000) for 15 min at RT and mounted with Vectashield mounting medium (Vector Laboratories). For negative controls, brain sections were stained with isotype control antibodies (Supplemental Fig. 2). Fluorescence images were captured with a Leica DMIRE2 inverted confocal laser-scanning microscope under the ×40 oil immersion objective lens. Samples were excited at 488, 543, and 633 nm and the emission fluorescence was recorded at 512–548, 556–650, or 650–750 nm, respectively. The image analysis was further described in supplementary methods.

### Flow cytometry

Anesthetized mice were transcardially perfused with 0.9% NaCl and the tumor mass tissues were identified and dissociated with the Neural Tissue Dissociation Kit per the manufacturer’s instruction (Miltenyi Biotech, Gladbach, Germany). Myelin was removed as per protocol published elsewhere^29^. Cells were dissolved in Hank’s Balanced Salt Solution containing FBS. For profiling of microglia, infiltrated macrophage, and myeloid-derived immune cells (TAMs), cells were stained with anti-mouse APC-CD11b, BV510-CD45, PE-Ym1, and eFluor 450-CD16/32 to assess TAM infiltration. For T cell profiling, cells were stained with PE-Cy5 CD8a, APC/Cy7-CD4, PE-FoxP3, APC-CD25, and APC-IFNγ. Intracellular staining of FoxP3 was done using intracellular staining buffer set (eBioscience) according to the manufacturer’s instruction. For detecting immune checkpoint blocker expression, cells were stained with PE-Cy5 CD8a, APC/Cy7-CD4, PE-PD-1, and PE-Cy7-CTLA-4. Samples were acquired with a BD LSRII instrument and analyzed with the Flow Jo (Tree Star) software.

### Statistical analysis

**Fig. 1:**
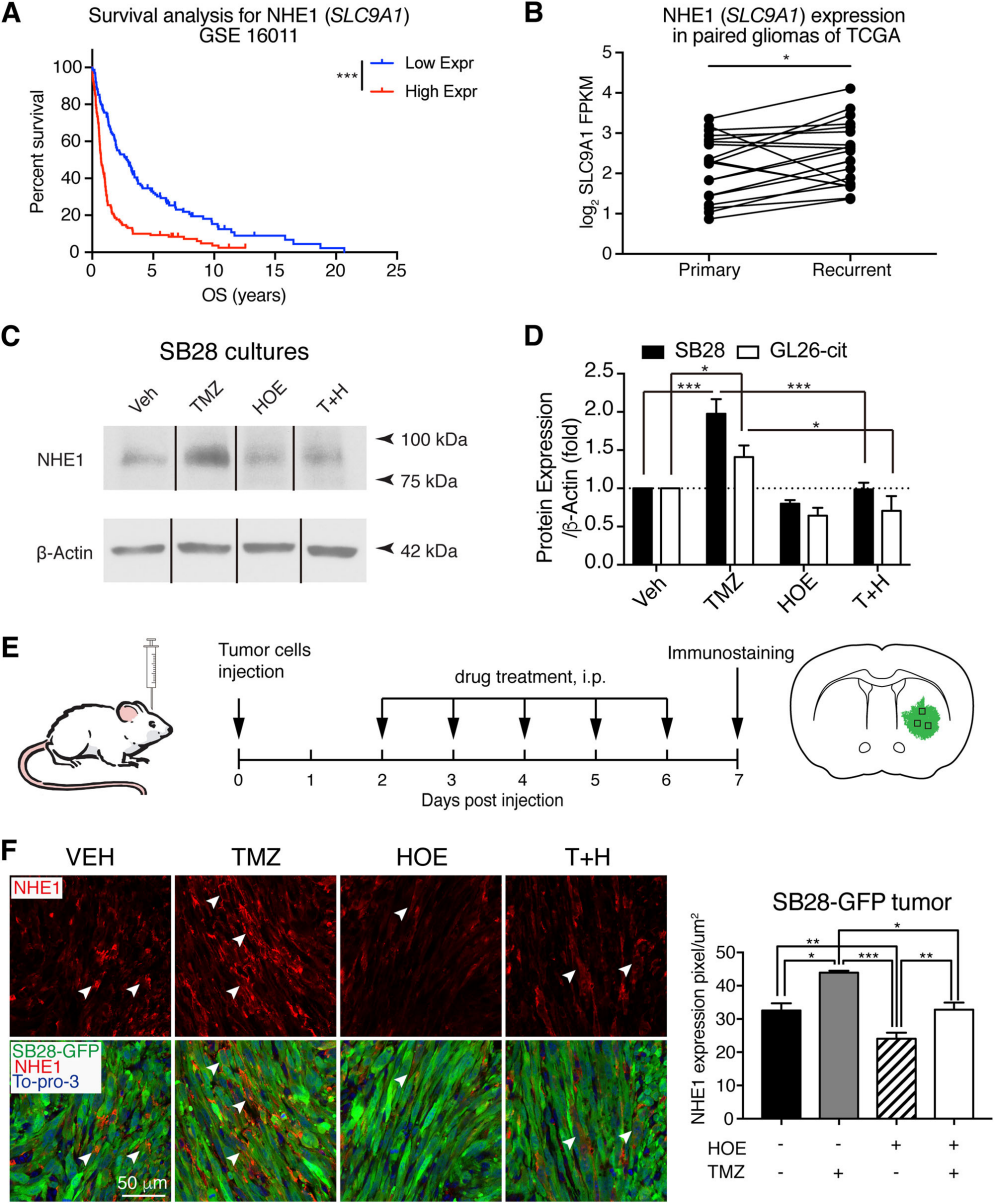
TMZ stimulates NHE1 expression in glioma. **a** Kaplan–Meier survival analysis of glioma patients with high NHE1 (*SLC9A1*) mRNA expression (*n* = 136) and low *SLC9A1* mRNA expression (*n* = 135) in CSE16011 cohort. ***p < 0.001. **b** *SLC9A1* gene expression in primary glioma (*n* = 20) and matched recurrent glioma (*n* = 20) were obtained from the RNA-seq data of TCGA dataset. *p < 0.05. **c**, **d** SB28-GFP cells or GL26-cit cells were exposed to TMZ (100 µM), HOE642 (1 µM), or combined for 24 h and cell lysates were harvested for immunoblotting of NHE1 protein. Data are means ± SEM from five independent experiments (*n* = 5), *p < 0.05, ***p < 0.001. **e** Experimental protocol and location of data collection. SB28 cells (50,000) or GL26-cit cells (40,000) were injected in the right striatum of C57BL6/J mice. Starting 2 days after implantation (d.p.i.), mice received either vehicle PBS-DMSO (10 ml/kg/day), TMZ (2.5 mg/kg/day), HOE642 (0.5 mg/kg/day), or T+H combination treatments (2.5, 0.5 mg/kg/day) for 5 consecutive days. Mice were sacrificed at 7 d.p.i. **f** Representative immunostaining of fixed brain sections (25 µm) for NHE1 protein expression in SB28-GFP tumors. Data are means ± SEM (*n* = 5). *p < 0.05, **p < 0.01, ***p < 0.001

**Fig. 2:**
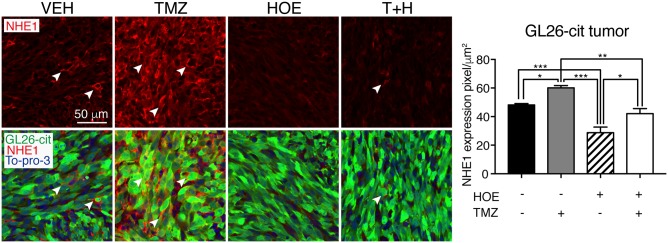
TMZ stimulates NHE1 protein expression in GL26-cit tumor. Representative immunostaining of fixed brain sections (25 µm) for NHE1 protein expression in GL26-cit tumor. Data are means ± SEM (*n* = 5). *p < 0.05, **p < 0.01, ***p < 0.001

**Fig. 3:**
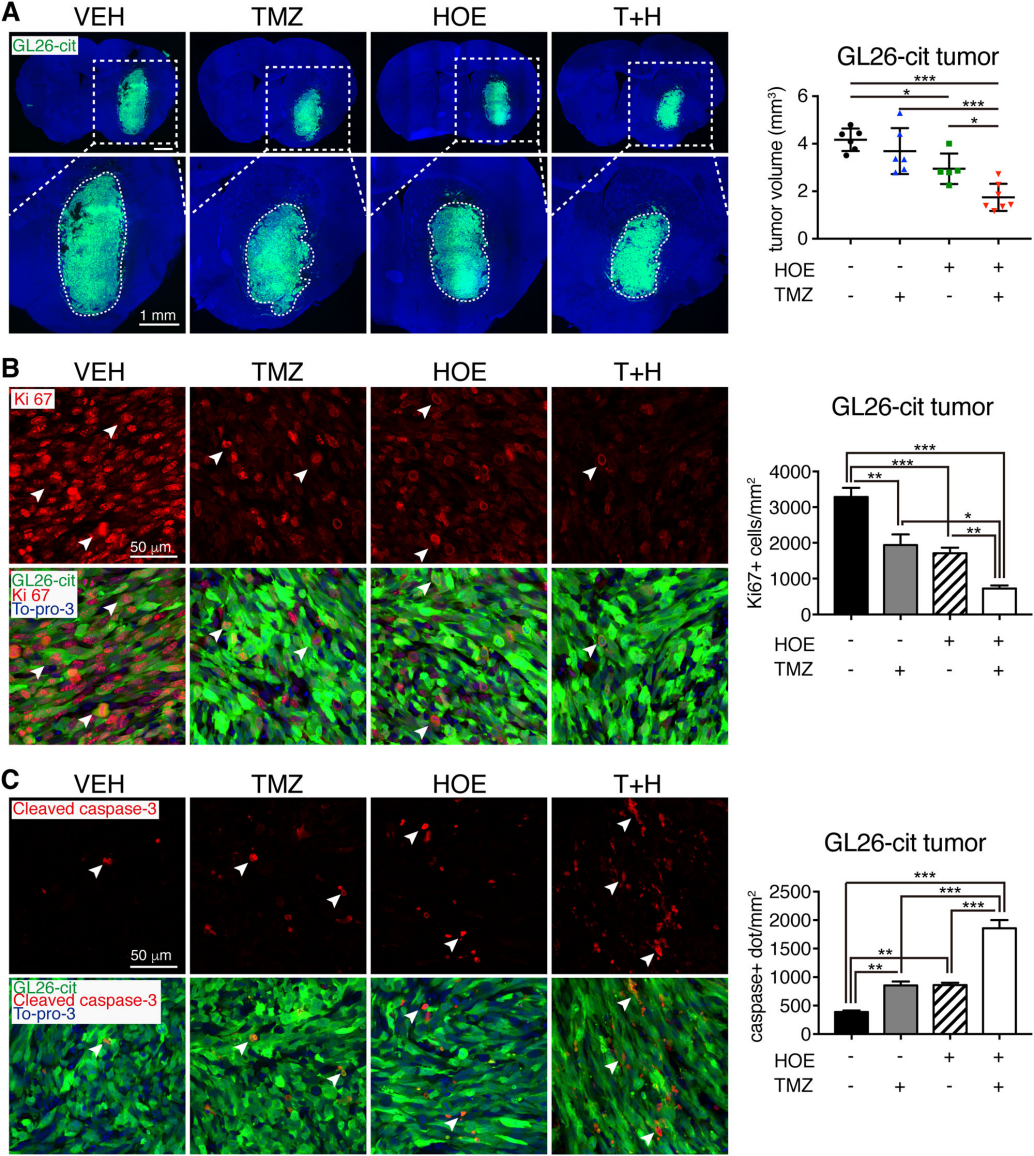
Combining pharmacological inhibition of NHE1 enhances TMZ therapy in inducing apoptosis and blockade of glioma growth. **a** Combination treatments reduced GL26-cit tumor volume at 7 d.p.i. in the same cohort of mice described in Fig. 1e. Data are means ± SD (*n* = 5–7). *p < 0.05, ***p < 0.001. **b** Ki67-positive proliferating cell counts in glioma tumors. Data are means ± SEM (*n* = 5). *p < 0.05, **p < 0.01, ***p < 0.001. **c** Cleaved caspase-3-positive apoptotic cell counts in glioma tumors. Data are means ± SEM (*n* = 5). **p < 0.01, ***p < 0.001

**Fig. 4:**
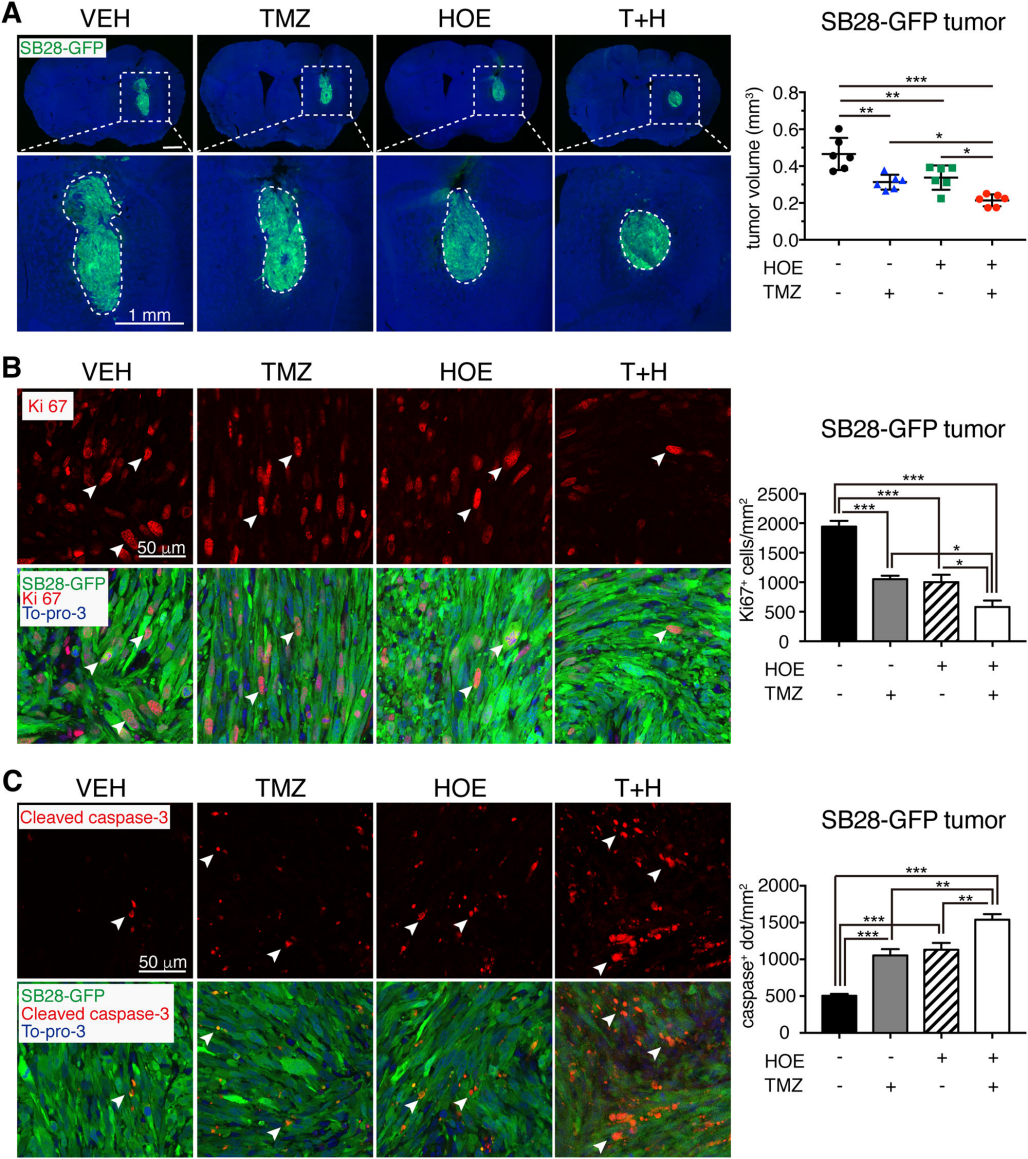
Combining pharmacological inhibition of NHE1 enhances TMZ therapy in inducing apoptosis and blockade of glioma growth. **a** Combination treatments reduced SB28-GFP tumor volume at 7 d.p.i. in the same cohort of mice described in Fig. 1e. Data are means ± SD (*n* = 6). *p < 0.05, **p < 0.01, ***p < 0.001. **b** Ki67-positive proliferating cell counts in GBM tumors. Data are means ± SEM (*n* = 5). *p < 0.05, ***p < 0.001. **c** Cleaved caspase-3-positive apoptotic cell counts in GBM tumors. Data are means ± SEM (*n* = 5). **p < 0.01, ***p < 0.001

**Fig. 5:**
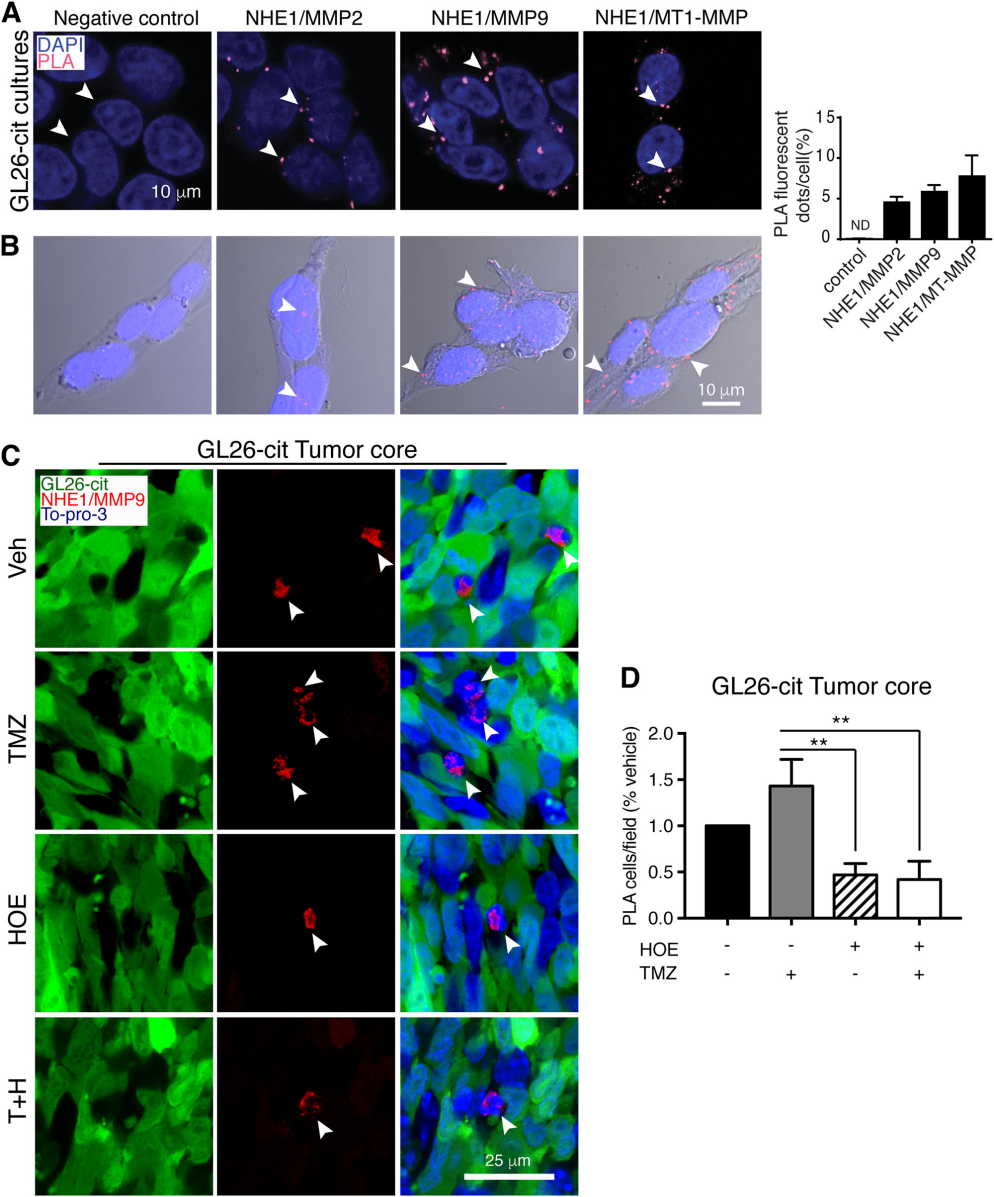
NHE1–MMPs interactions in glioma tumors. **a** Proximity ligation assay (PLA) for NHE1-MMP2, NHE1-MMP9, or MT1-MMP interactions in GL26-Cit cells in cultures. Data are expressed as the number of fluorescence dots/cell (arrowhead). Data are means ± SEM (*n* = 4–5). Scale bar, 10 μm. **b** Representative confocal images of DAPI and PLA merged with DIC (Differential interference contrast) images for NHE1-MMP2, NHE1-MMP9, or MT1-MMP interactions (arrowhead) in GL26-Cit cells in cultures. **c** Representative confocal images of GFP and PLA (NHE1-MMP9) immunofluorescence signals (arrowhead) in the tumor core. **d** Summary data of PLA-positive cells were expressed as the percentage of vehicle control. Data are mean ± SEM (*n* = 6–7). **p < 0.01

**Fig. 6:**
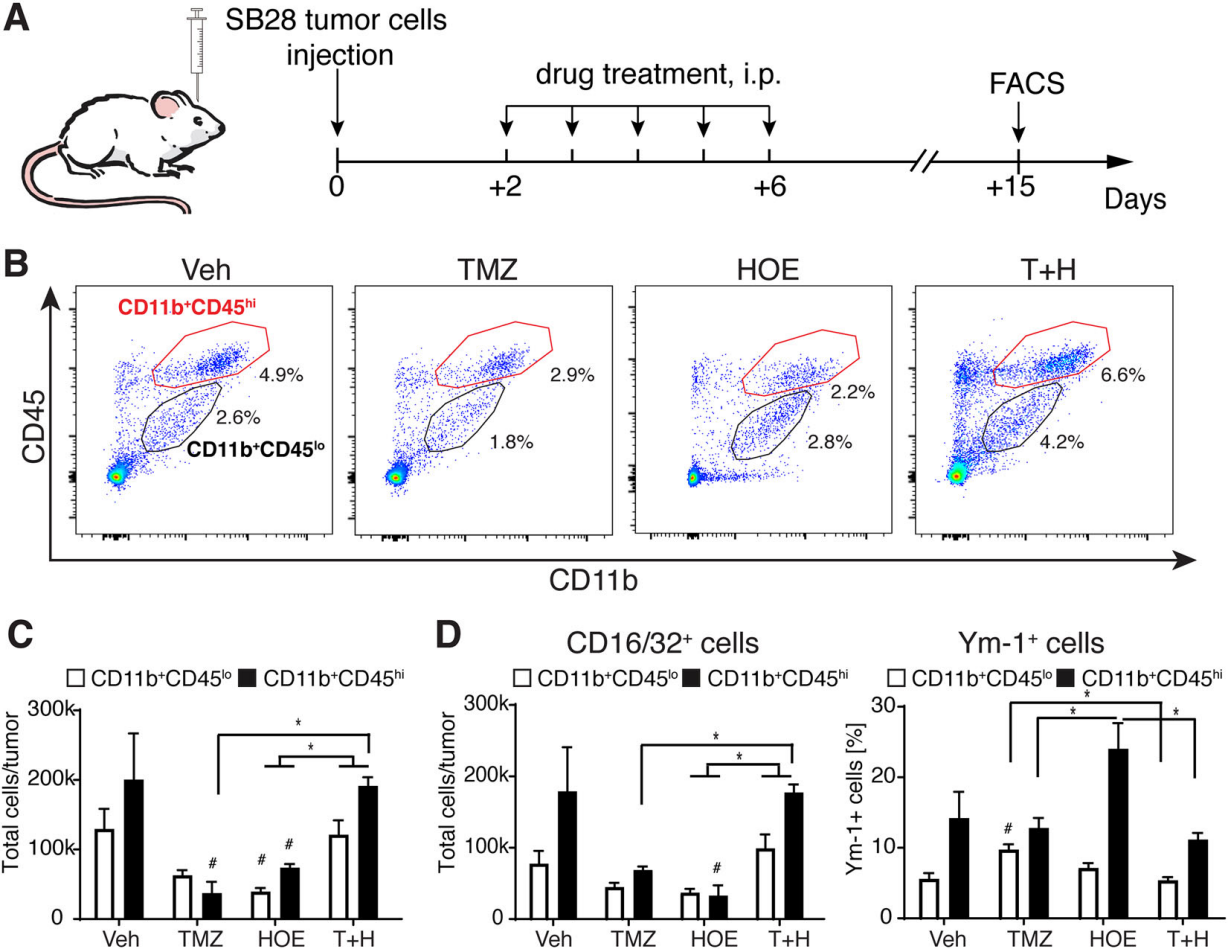
NHE1 blockade in combination with TMZ treatment increases antitumor pro-inflammatory TAMs. **a** Starting 2 d.p.i. of SB28 cells implantation, mice received either vehicle PBS-DMSO, HOE642, TMZ, or T+H combination treatments for 5 consecutive days. Mice were sacrificed at 15 d.p.i. and flow cytometric analysis of tumor tissues was performed. **b** Representative flow cytometric profile showing gating strategy of microglia (CD11b^+^/CD45^low-medium^) and infiltrating myeloid cells (CD11b^+^/CD45^hi^). **c** Total number of CD11b^+^/CD45^low-medium^ and CD11b^+^/CD45^hi^ cells. **d** Inflammatory profile of CD11b^+^/CD45^low-medium^ and CD11b^+^/CD45^hi^ cells. All data are mean ± SEM, *n* = 5–6, ^#^*p* ≤ 0.05 vs. Veh-control, *p ≤ 0.05

**Fig. 7:**
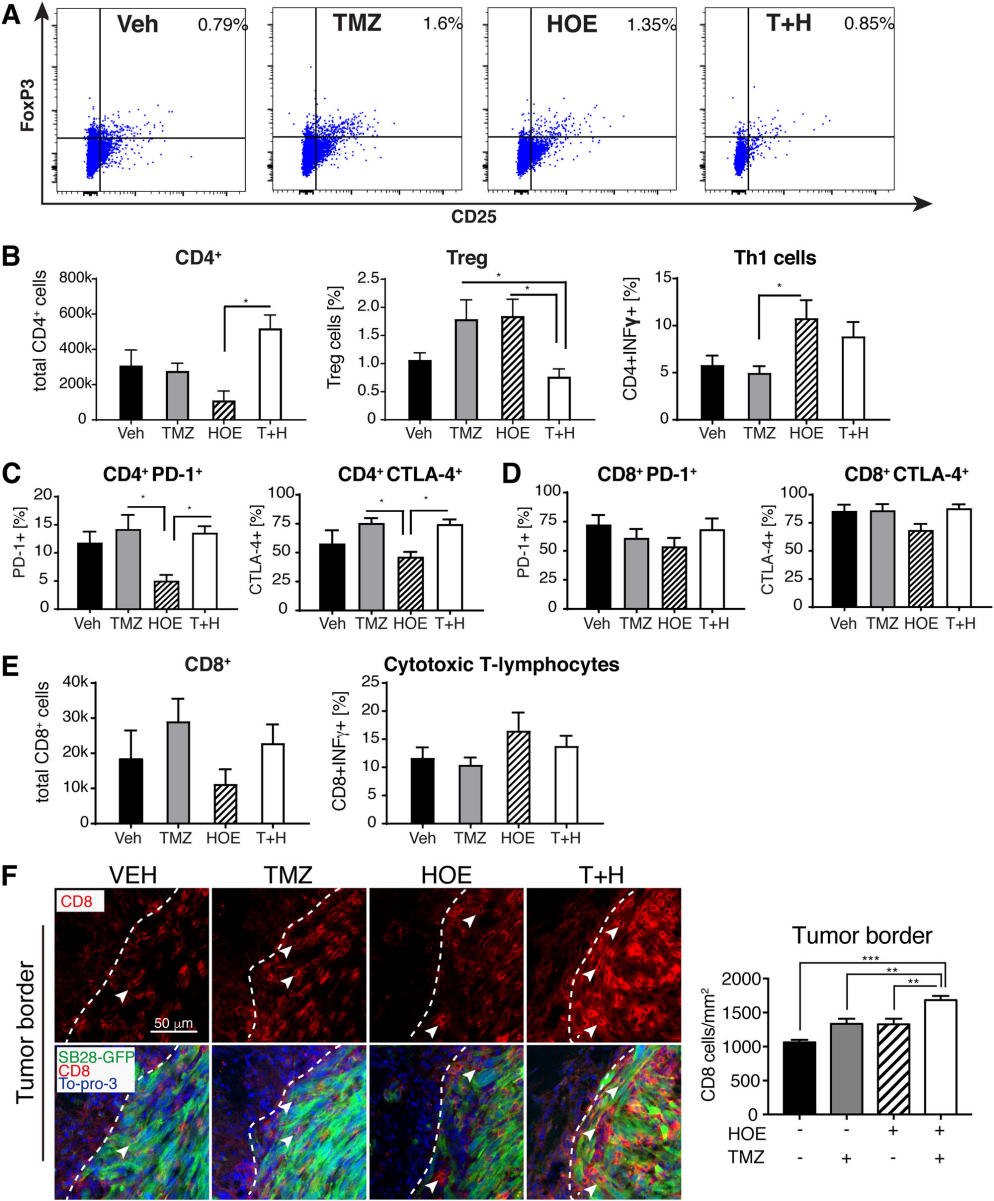
Blockade of NHE1 enhances the T cell antitumor immunity in glioma. **a** Representative flow cytometric profile of CD4^+^CD25^+^FoxP3^+^ (Treg). **b** Total CD4^+^ T cell counts and the percentage of CD4^+^IFNγ^+^ and Treg cells within CD4^+^ population. **c** Percentage of PD-1 and CTLA-4 expression in CD4^+^ T cell population. **d** Percentage of PD-1 and CTLA-4 expression in CD8^+^ T cell population. **e** Total CD8^+^ T cell counts and the percentage of IFNγ^+^ cells in CD8^+^ population. Data are mean ± SEM (*n* = 5–7). **f** Infiltration of CD8^+^ T cells (arrowhead) in SB28-GFP tumor borders (dashed line). Data are mean ± SEM (*n* = 5). *p ≤ 0.05, **p < 0.01, ***p < 0.001

**Fig. 8:**
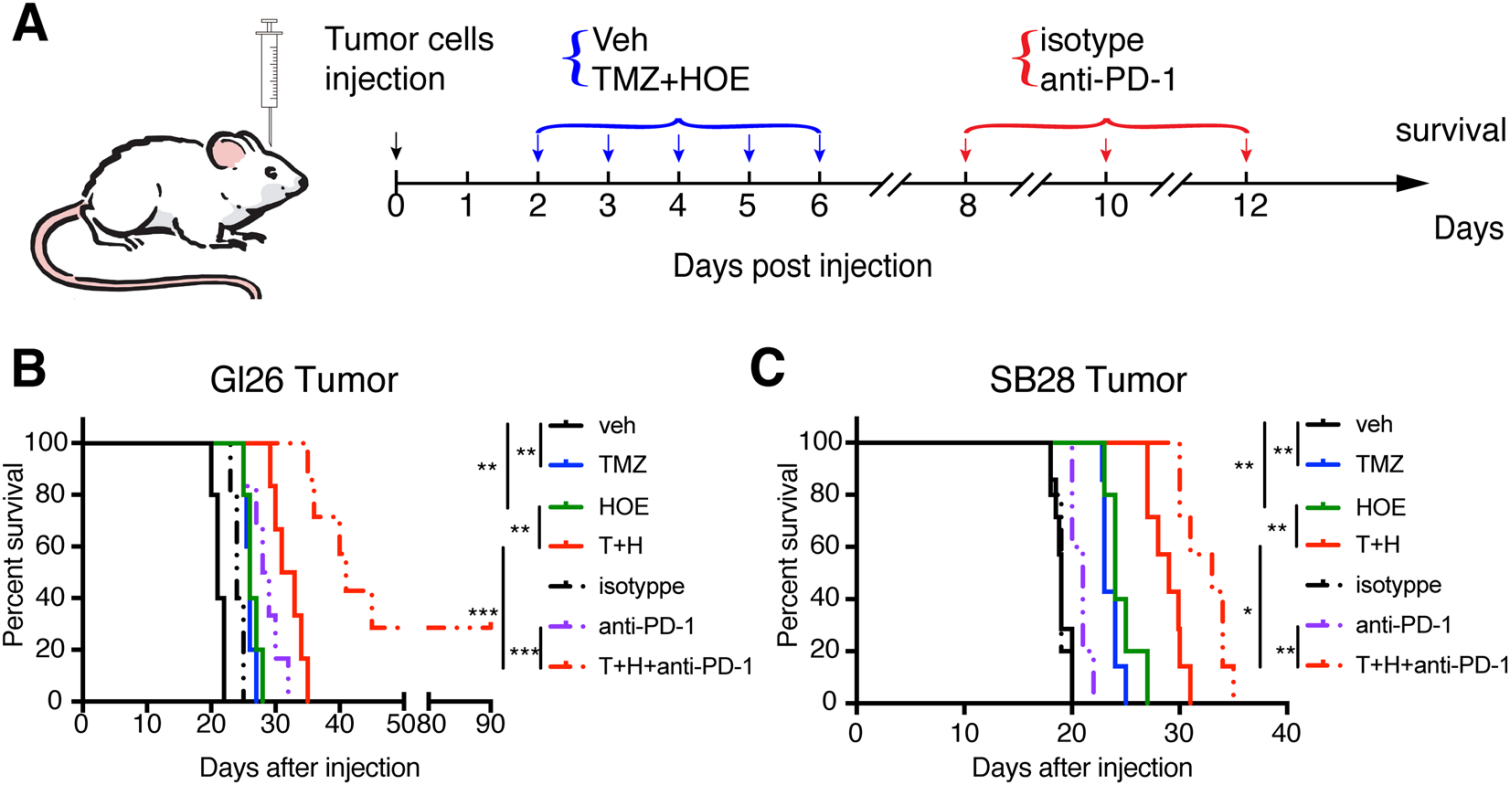
The combinatorial regimen of HOE642, TMZ, and anti-PD-1 antibody increases glioma-bearing mouse survival. **a** Experimental protocol and location of data collection. Glioma cells were injected into the right striatum of C57BL6/J mice. Starting 2 d.p.i., mice received vehicle PBS-DMSO (10 ml/kg/day, i.p.), TMZ (2.5 mg/kg/day, i.p.), or T+H combination treatments (2.5+0.5 mg/kg/day, i.p.) for 5 consecutive days. Then mice received either isotype antibody (10 ml/kg/day, i.p.) or anti-PD-1 (10 mg/kg/day, i.p.) at 8, 10, and 12 d.p.i. **b** Kaplan–Meier survival curve of GL26 tumor-bearing mice (each group *n* = 5–7). **p < 0.01, ***p < 0.001. **c** Kaplan–Meier survival curve of SB28-GFP tumor-bearing mice (each group *n* = 5–7). **p < 0.01, ***p < 0.001

The results were expressed as the mean ± standard error of the mean (SEM) or standard deviation (SD). Using Prism 7 (GraphPad Software, Inc), statistical significance was determined by paired *t* test for matched groups (Fig. 1b) or analysis of variance followed by Bonferroni’s multiple comparison test for multiple comparisons (Figs. 1d, f, 2, 3, 4, 5, 6c, d, and 7b–e). Overall survival of patients or mouse median survival time was evaluated by using Kaplan–Meier analysis and compared with a two-sided log-rank test (Figs. 1a and 8). A *p* value <0.05 was considered statistically significant. *N* values represent the number of in vitro or in vivo experiments.

## Results

### Elevated NHE1 mRNA expression in human glioma tissues

To better understand the role of NHE1 in the progression of glioma, we compared the overall survival time of glioma patients between the group with high and low NHE1 (*SLC9A1*) mRNA expression in the GSE16011 cohort. Kaplan–Meier survival curve showed that the patients with high level of NHE1 mRNA in glioma tissues had a shorter overall survive (*p* < 0.001, Fig. 1a). Moreover, we surveyed NHE1 gene expression in primary and recurrence glioma patients, and the matched recurrent glioma showed higher NHE1 mRNA expression than primary glioma (*p* < 0.05, Fig. 1b). These findings clearly suggest that NHE1 protein is involved in tumor progression and support the premise of this study.

### TMZ stimulates NHE1 protein expression in mouse glioma tumors

Intracranial allografts of GL26-cit glioma cells represent a well-established mouse syngeneic glioma model^23,30^. SB28-GFP is a newly developed mouse cell line, which does not express detectable CD40, and represents a weakly immunogenic glioma model^24^. NHE1 protein was expressed in both SB28-GFP and GL26-cit glioma cultures (Fig. 1c, d). In response to TMZ (T) treatment for 24 h, NHE1 protein levels were elevated by 41–91% (in GL26 and SB28 cells, respectively, *p* < 0.05). Treatment of glioma cells with NHE1 inhibitor HOE642 (H) alone had no effects on its expression, but the combined treatment (T+H) abolished the TMZ-induced elevation of NHE1 protein.

Increased NHE1 protein promotes glioma survival and we expected that blocking NHE1 function with NHE inhibitor HOE642 would sensitize glioma cells to TMZ-induced cytotoxicity. As illustrated in Fig. 1e, C57BL/6J mice were transplanted with SB28-GFP or GL26-cit cells and mice received four treatment regimens. NHE1 protein levels were low in tumors in the Veh-control mice (arrowhead, Fig. 1f), which was further reduced in SB28-GFP tumors treated with HOE642. In contrast, SB28 tumor-bearing mice treated with TMZ displayed a significant increase in NHE1 protein expression (20 ± 6%, *p* < 0.05, Fig. 1f). Consistently, administration of the T+H treatment regimen blocked TMZ-mediated effects (Fig. 1f). Similar to the SB28-bearing tumors, TMZ induces NHE1 protein upregulation in GL26-cit tumors and combination of T+H prevented the TMZ-induced NHE1 protein elevation (Fig. 2).

### Combined TMZ treatment with NHE1 inhibition reduces tumor growth via inhibiting proliferation and enhancing apoptosis in mouse glioma allografts

We then evaluated whether inhibition of NHE1 protein sensitizes tumors to TMZ-mediated toxicity in the mouse glioma models. Compared with the Veh-control, combining TMZ therapy with blockade of NHE1 decreased both GL26-cit (Fig. 3a) and SB28-GFP glioma growth (Fig. 4a). The order of tumor volume was the following: Veh-control > TMZ > H or T+H (*p* < 0.05**)**. The immunoreactive intensity of Ki67, a marker for activated cell cycle^31^, was high in the GL26-cit tumor cells in the Veh-control mice (Fig. 3b). HOE642 or TMZ monotherapy decreased Ki67^+^ tumor cells by ~48.0% or 41.1%, respectively (*p* < 0.01). The T+H combination group further reduced Ki67^+^ cells in GL26-cit glioma tumors by 78.0% (*p* < 0.001). An identical pattern of the changes in Ki67^+^ cells was detected in the SB28-GFP glioma tumors (Fig. 4b).

Moreover, either blocking NHE1 protein with HOE642 or TMZ monotherapy triggers glioma tumor apoptosis increases the cleaved caspase-3 expression by ~2.2-fold in GL26-cit tumors (*p* < 0.01, Fig. 3c). Combining TMZ with HOE642 further stimulated cleaved caspase 3 activation in GL26-cit tumors (~4.8-fold, *p* < 0.001). TMZ combined with HOE642 also increased caspase 3 cleavage in the SB28-GFP glioma tumors (Fig. 4c). These results clearly indicate that combining TMZ with HOE642 is effective in decreasing glioma volume via inhibition of proliferation and accelerating apoptosis in the mouse intracranial syngeneic models of glioma.

### NHE1-MMPs interactions in glioma tumors

We hypothesize that NHE1 protein provides acidic extracellular microenvironment to activate matrix metalloproteinases (MMPs) in the expansion of malignant gliomas. To test this possibility, we conducted PLA to determine whether NHE1 is closely located with MMP2, MMP9, or MT1-MMP in glioma cells. Negative control staining, by omitting one of the primary antibodies, did not exhibit any fluorescence signals in cultured GL26-cit cells (Fig. 5a). In contrast, bright punctate fluorescence signals (red) were detected when the cells were incubated with the anti-NHE1 and the anti-MMP2 antibodies (4.1 ± 0.7 PLA dots/cell), the anti-MMP9 antibodies (6.2 ± 1.1 dots/cell), or with the anti-MT1-MMP antibodies (7.8 ± 2.6 dots/cell, Fig. 5b). These data suggest that NHE1 protein is closely located with MMP2, MMP9, and MT1-MMP proteins in GL26-Cit tumors under basal conditions.

In the GL26-cit syngeneic glioma tumor model, PLA NHE1-MMP9 signals were detected in the tumor core area with tightly packed GFP^+^ tumor cells (arrowhead, Fig. 5c). Compared to the Veh control, TMZ monotherapy increased NHE1-MMP9 PLA signals in the tumor core regions, but it is not statistically significant. However, HOE642 treatment or the T+H combination treatment nearly abolished NHE1-MMP9 PLA clustering signals in the core regions (*p* < 0.05, Fig. 5d). PLA NHE1-MMP9 signals were also detected in the tumor border areas (at the edges of tightly packed GFP^+^ tumor cells, Supplemental Figure 3), but these signals were absent in the tumors treated by HOE642 or the T+H combination. However, these PLA signals were not overlapped with the GFP signals, suggesting that they are non-tumor cells. Taken together, these findings imply that NHE1 may regulate glioma progression via closely regulating MMP expression and activation.

### NHE1 blockade in combination with TMZ polarizes inflammatory stimulation of TAMs and regulatory T cells

We further investigated the effect of NHE1 blockade in combination TMZ treatment on glioma tumor microenvironment. C57BL/6 mice were transplanted with SB28 glioma cells and treated with Veh, HOE642, TMZ, or T+H regimens, and flow cytometric analysis of infiltrated TAM and T cells was conducted (Fig. 6a). Based on CD11b and CD45 expression levels, microglia (CD11b^+^/CD45^low-med^) and CD11b^+^/CD45^hi^ population were defined in the flow cytometric scatter plot (Fig. 6b). The microglia population was further confirmed by characterization for their expression of selective marker protein P2RY12 (~88% CD11b^+^/CD45^low-med^ population are P2RY12^+^ cells, data not shown). Considering infiltrated CD11b^+^/CD45^hi^ cell population in tumors contains not only macrophages but also activated, immature myeloid-derived suppressor cells (such as CD11b^+^/Gr-1^+^ cells)^32,33^, we refer CD11b^+^CD45^hi^ cell population as infiltrating myeloid cells. This view is further supported by our findings that 72% of CD11b^+^/Gr-1^+^ cells overlapped with CD11b^+^/CD45^hi^ population (Supplemental Figure 4). SB28 glioma tumors contained abundant amounts of microglia (CD11b^+^/CD45^low-med^) and infiltrating myeloid cells (CD11b^+^/CD45^hi^) (Fig. 6b, c). HOE642 and TMZ monotherapy reduced microglia (CD11b^+^/CD45^low-med^) and infiltrated myeloid cell (CD11b^+^/CD45^hi^) numbers by ~47 and 53%, respectively (*p* < 0.05, *p* = 0.1, Fig. 6c). In contrast, the T+H treatment caused ~2.5-fold increase in microglia/infiltrated myeloid cell numbers (*p* < 0.05, Fig. 6c). HOE642 or TMZ monotherapy also reduced CD16/32^+^ proinflammatory cells (*p* < 0.05 and *p* = 0.1 respectively, Fig. 6d). Interestingly, T+H treatment elevated CD16/32^+^ proinflammatory TAMs by ~2.5-fold and concurrently reduced Ym-1^+^ anti-inflammatory cells by ~2-fold (*p* < 0.05, Fig. 6d). We further investigated the impact of the combination therapy on the glioma microenvironment in GL26 tumors. T+H treatment caused ~30% increase in CD11b^+^/CD45^hi^ population, compared to HOE642 or TMZ monotherapy, whereas CD11b^+^/CD45^low-medium^ population was unchanged (Supplemental Figure 5A-C). T+H treatment triggered ~2-fold increase in CD16/32^+^ proinflammatory cells (Supplemental Figure 5D).

To investigate potential effects of blocking NHE1 on immune cells from non-tumor brain tissues, we compared immune cell profiles from contralateral hemisphere non-tumor tissues (CL) and ipsilateral hemisphere tumor tissues (IL). The results show that no changes of either CD11b^+^/CD45^low-medium^ population or CD11b^+^CD45^hi^ population were observed in the CL hemisphere non-tumor tissues with or without the drug treatments (Supplemental Figure 6). In contrast, the IL tumor tissues exhibited higher number of either CD11b^+^/CD45^low-medium^ population or CD11b^+^CD45^hi^ population, which was further increased in response to TMZ or TMZ+HOE642 treatment (Supplemental Figure 6). These findings suggest that the immune cells of the tumor tissues are specifically responsive to TMZ or TMZ+HOE642 treatment.

We also investigated tumor-infiltrated T cell profiles in SB28 glioma tumors under four treatment regimens. CD4^+^CD25^+^FoxP3^+^ cells were identified in a flow cytometric scatter plot (Fig. 7a). CD4^+^ T cells infiltrated into the SB28 glioma tumors in the Veh-control mice (Fig. 7b). HOE642 monotherapy increased regulatory T (Treg) population (CD4^+^CD25^+^FoxP3^+^) by ~100% and T helper type 1 (Th1) (CD4^+^IFNγ^+^) population by ~ 80% (*p* = 0.29 and *p* = 0.18, respectively). TMZ monotherapy moderately elevates Treg population, which did not reach statistical significance (*p* > 0.05). Interestingly, in the case of T+H therapy, an increase in infiltration of CD4^+^ T cells and Th1 cells and a decrease in Treg cells were detected (*p* < 0.05, Fig. 7b). There were no significant changes in the expression of immune checkpoint blockers, PD-1 and CTLA-4, in either CD4^+^ or CD8^+^ T cells (Fig. 7c, d). In the case of GL26 glioma tumors, T+H treatment triggered ~20–50% increase in CD4^+^ T cell infiltration, compared to TMZ or HOE642 monotherapy alone (Supplemental Figure 7). PD-1 expression in CD4^+^ or CD8^+^ T cells of GL26 tumors was lower than in SB28 tumors, but no differences in PD-1 expression were detected among the treatments (Fig. 7c, Supplemental Figure 7).

To further investigate the impact of blockade of NHE1 function in immunogenicity in glioma tumors, we conducted immunostaining assay for CD8^+^ T cells to evaluate immune cell infiltration in the tumor. CD8^+^ cells infiltrated into SB28 tumors and were accumulated at the tumor border (Fig. 7e). HOE642 or TMZ monotherapy did not significantly change the CD8^+^ infiltration. In contrast, the T+H combination regimen significantly increased CD8^+^ infiltration both at the tumor core and tumor border, especially at the latter by ~60% (*p* < 0.05, Fig. 7e and Supplemental Figure 8). Taken together, these data clearly show that NHE1 blockade in combination with TMZ treatment alters glioma microenvironment via stimulating pro-inflammatory polarization of TAMs, increased T cell infiltration and the cytotoxic T cell activation, and decrease of Treg cell population.

### Combing blockade of NHE1 with TMZ and anti-PD-1 therapy significantly increases survival of glioma-bearing animals

We then conducted a survival study in a cohort of mice that were randomly assigned for the mono or combinatorial treatment regimens and monitored mice until they reached the humane end point (Fig. 8a). In the case of immunogenic GL26 glioma tumor model, HOE642 or TMZ treatment extended the median survival to ~26 days (*p* < 0.01, Fig. 8b). The Veh (isotype antibody) control group exhibited similar median survival period (~24 days, Fig. 8b). The T+H treatment or the anti-PD-1 monotherapy further prolonged the medium survival time (~32 and 28.5 d.p.i, *p* < 0.05). Interestingly, the combinatorial therapy of T+H followed by 3 days of anti-PD-1 antibody extended the median survival to ~41 d.p.i. (*p* < 0.05, Fig. 8b), especially ~30% mice survived for 90 d.p.i. In the SB28-GFP tumor-bearing mice, the Veh (DMSO) control, the Veh control (isotype antibody), or the anti-PD-1 antibody group exhibited a short median survival time (~19, ~19 days, and ~21 days, Fig. 8c). HOE642 or TMZ treatment extended the median survival to ~24 or ~23 days, respectively (*p* < 0.01). T+H treatment further prolonged the medium survival to ~29 d.p.i (*p* < 0.05, Fig. 8c). T+H treatment, which was followed up with 3 days of anti-PD-1 treatment, increased the mouse median survival to ~33 d.p.i (*p* < 0.05, Fig. 8c). Taken together, these findings allow us to conclude that combining pharmacological blockage of NHE1 with TMZ therapy improves TMZ monotherapy. Blocking NHE1 protein also improves immunosuppressive microenvironment and sensitizes glioma tumors to checkpoint blockade immunotherapy.

## Discussion

### TMZ-induced upregulation of NHE1 protein in glioma

The GSE16011 dataset shows that the patients with high NHE1 mRNA expression are associated with poor overall survival. In addition, recurrent gliomas presented higher levels of NHE1 mRNA than primary gliomas. A major role of NHE1 in cells is to maintain pH_i_ and cell volume homeostasis. We previously observed that TMZ stimulates NHE1 protein expression in cultured human primary glioma cells^17^. The elevated NHE1 protein supports alkaline pH_i_ and prevents apoptotic volume decrease^34^. In this in vivo study, TMZ treatment for 5 consecutive days increased NHE1 protein expression in glioma cells by ~20%. Interestingly, the TMZ-mediated upregulation of NHE1 expression was inhibited by combinatorial regimen of TMZ+NHE1 inhibitor HOE642.

The signaling events that regulate NHE1 expression and function in tumor cells are not well understood. The tumor microenvironment is characterized as hypoxic and acidic^35,36^. Hypoxia not only contributes to tumor formation but is also involved in developing chemoresistance in GBM^37^, in part through activating hypoxia-inducible factor-1 alpha (HIF-1α) expression and its downstream signaling pathways. Promoter region of NHE1 gene expresses HIF-1-binding site^38^. TMZ treatment downregulates HIF-1α mRNA expression in the TMZ-sensitive glioma cells (U251 and U87) but upregulates the HIF-1α mRNA expression in the TMZ-resistant glioma cells (T98 and U138)^39^. In addition, elevated HIF-1α protein mediates GBM resistance to TMZ by regulation of MGMT transcription^40^. Knockdown of HIF-1α expression increases sensitivity of glioma to TMZ treatment^39,41,42^. Thus TMZ-mediated stimulation of HIF-1α activity may be responsible for upregulating NHE1 expression in glioma. Additional studies are needed to investigate mechanisms underlying HIF-1α-mediated NHE1 expression and its impact on the TMZ chemoresistance in glioma. In addition, the epidermal growth factor (EGF) and Rho-associated protein kinase are also involved in regulating expression of NHE1 in multiple pancreatic ductal adenocarcinoma cell lines^43^ and in DU145 prostate cancer cells^44^. EGF receptor plays an important role in glioma progression and TMZ resistance by activating Ras/Raf/mitogen-activated protein kinase (MAPK) or phosphoinositide-3 kinase/AKT/mammalian target of rapamycin signaling^45^. Whether these molecules are responsible for TMZ-induced increase of NHE1 protein expression in glioma cells remain to be determined.

TMZ+HOE642 combination treatment reduced NHE1 protein expression in SB28 or GL26 gliomas. The mechanisms underlying effects of HOE642 (Cariporide) on NHE1 protein expression are not clear. Cariporide treatment has been shown to reduce NHE1 mRNA and protein expression in isolated rat renal cortex^46^. Glioma hypoxic microenvironment and acidosis stimulate the HIF-1α expression^47,48^, which may result in upregulation of NHE1 expression and promote the acidic microenvironment^49–51^. Therefore, inhibition of NHE1 activity with HOE642 could relieve the acidic, hypoxic microenvironment and reduce HIF-1α expression, leading to the downregulation of NHE1 protein expression. Other factors could also play a role in regulating NHE1 protein expression. It has been reported that ERK1/2 regulates NHE1 protein expression and activity via p38 MAPK signaling pathway^52^. NHE1 protein directly interacts with extracellular signal–regulated kinase 1/2 (ERK1/2) and functions as a scaffold protein in stimulating ERK activity^53^, and knockdown of NHE1 or inhibition of NHE1 with cariporide reduces ERK1/2 expression in human breast cancer cell^54^. Thus inhibition of NHE1 protein with HOE642 could modulate NHE1 protein expression via these mechanisms, which need to be further investigated.

### Combination treatment is better than TMZ therapy on reducing tumor growth and tumor volume

We found that the combination therapy with TMZ+HOE642 reduced the tumor volume of mice bearing either GL26-cit tumor or SB28-GFP tumor. In addition, the combination therapy decreased tumor proliferation and concurrently enhanced TMZ-mediated apoptosis. Treatment of mice with the T+H combination enhanced the TMZ therapeutic effect on inhibiting tumor growth by ~57% in GL26 tumor and ~45% in SB28-GFP tumor. To our knowledge, the current study is the first to demonstrate the role of NHE1 in glioma growth of syngeneic glioma animal models.

The role of pH_i_ has been verified in regulation of cell proliferation and growth for several decades^55^. A pH_i_ > 7.2 promotes cells to enter S phase under simulation of growth factor and progress through the G2/M phase^56^. NHE1, as an important regulator of pH_i_, was also assigned a central role in the G2/M progression via the regulation of pH_i_^50^. Additionally, NHE1 protein may facilitate tumor proliferation by specific regulation of cell cycle kinases to support DNA synthesis, protein synthesis, and cell metabolism^50^.

### Blocking NHE1 alone or combination therapy is effective in reducing tumor progression and improving survival

In our previous study, inhibition of NHE1 expression decreased glioma cell migration in vitro^17^. We have also shown that NHE1 protein is colocalized with ezrin protein in glioma cell lamellipodia and plays a role in glioma migration through regulating changes of cytoskeletal structures^17^. Glioma invasion occurs via secretion of MMPs into tumor microenvironment and degradation of extracellular matrix^57^. The MMP protein family members are vital for tumor invasion. Our PLA study revealed close localization of NHE1 and MMP-9 in glioma tumors, which could promote invasion/proliferation of glioma cells via activating MMPs. We also detected strong PLA signals in non-tumor cells in the tumor core areas or border regions. MMP9 has been shown to be predominantly expressed in TAMs in glioma tissues but not the glioma cells^58^. In addition, myeloid-derived granulocytes and monocytes are also the major source of MMP9 expression in gliomas tissues^59,60^. It has been suggested that MMP9 released from these cells promotes angiogenesis and tumor progression. In our study, TMZ treatment increased the formation of NHE1–MMP9 complex in non-glioma cells in both tumor border and core areas. However, treatment with HOE642 or the combination T+H therapy prevented increase of NHE1 protein expression and reduced formation of NHE1 and MMP9 complex. Collectively, the reduced NHE1 protein expression and less NHE1–MMP9 complex formation could play a role in decreasing immunosuppression and inhibition of tumor progression.

### Blockade of NHE1 stimulates pro-inflammatory polarization of TAMs and promotes antitumor immunity

The tumor microenvironment plays an important role in the progression of tumors^61^. In addition to tumor cells, gliomas contain non-neoplastic cells, including astrocytes, monocytes, and immune cells^62^. A total of 30–50% of the cells in gliomas are TAMs, which provide protumoral microenvironment^63^. In this study, we found that combining blockade of NHE1 with TMZ therapy stimulated the CD16/32^+^ pro-inflammatory TAMs in SB28 glioma tumors. Meanwhile, combination treatment of HOE642 and TMZ reduced Ym-1^+^ anti-inflammatory cells. Conversion of anti-inflammatory M2 TAMS to inflammatory M1 phenotype or increase in M1 TAMs number has been suggested as a novel antitumor therapeutic strategy^63^. Our findings suggest that combining inhibition of NHE1 with TMZ therapy stimulates pro-inflammatory M1 TAMs and reduces anti-inflammatory M2 TAMs. The similar pattern was also detected in GL26 glioma tissue.

Cancer immunotherapy targets the regulation of T cells to enhance the antitumor immunity and has achieved the success against certain cancers^15^. However, clinical trials for GBM patients with different vaccines show no responses or only 30% were responsive^8,9,64^. In part, this may result from a non-immunogenic tumor microenvironment, which lacks the expression of immunogenic markers or infiltration of immune cells into tumors^15^. Thus combination treatment is essential to improve the immunogenicity of glioma microenvironment. In this study, combination treatment of T+H increased CD8^+^IFNγ^+^ cell infiltration and reduced Treg cells and especially promoted the infiltration of CD8^+^ cells into the weakly immunogenic SB28-GFP tumors. In addition, combination treatment with NHE1 blockade, TMZ, and the anti-PD-1 immune checkpoint therapy prolonged the glioma-bearing mouse median survival in both SB28-GFP and GL26 glioma models. However, it did not achieve better outcome (only 2/7 mice lived >90 days d.p.i.). The causes could be due to less optimal doses or treatment timing. HOE642 displays a short half-life (~3.5 h in human serum^65,66^), and further optimizing the combination therapy protocol by concurrent administration of HOE642 with the anti-PD-1 therapy or optimizing dosages of HOE642 should be considered in future experimental designs.

In summary, we report here that TMZ therapy increases the expression of cytoprotective protein NHE1 in glioma. Combining TMZ therapy with NHE1 inhibitor enhances TMZ-induced glioma apoptosis and reduces proliferation and tumor growth. Moreover, blockade of NHE1 stimulates pro-inflammatory polarization of TAMs and increases tumor infiltration of CD8^+^ cells. Furthermore, combining inhibition of NHE1 with TMZ and anti-PD-1 therapy significantly extends the median survival in both immunogenic and non-immunogenic mouse glioma models (Supplemental Figure 9). Our findings suggest that blockade of NHE1 protein presents a new strategy for improving TMZ chemotherapy and antitumor immunotherapy.

## Electronic supplementary material

Supplementary file
