# Supplementary material for: Blockade of Na/H exchanger stimulates glioma tumor immunogenicity and enhances combinatorial TMZ and anti-PD-1 therapy
Source: Cell Death Dis. 2018 Sep 27;9(10):1010. doi: 10.1038/s41419-018-1062-3 (PMC6160445; doi:10.1038/s41419-018-1062-3)
Supplement: Supplementary file 1 — Supplementary file [file 41419_2018_1062_MOESM1_ESM.docx]

**Supplemental Information**

**Supplemental Materials and Methods**

**Materials**: Cariporide (HOE-642) and TMZ were purchased from Sigma Chemicals (St. Louis, MO). Dulbecco’s Modified Eagle Medium (DMEM/HEPES, Cat #12430-054) and Penicillin/streptomycin (Cat #15240062) were from Gibco (Carlsbad, CA). Fetal bovine serum (FBS) and G418 sulfate were obtained from Invitrogen (Carlsbad, CA). Anti-mouse PD-1 (Cat #BE0146) and isotype IgG2a were from BioXCell (West Lebanon, NH). Rabbit antibody against Ki67 (Cat #9129S), rabbit antibody against cleaved caspase-3 (Cat #9661S) and rabbit antibody against β-actin (Cat #4970S) were from Cell Signaling (Beverly, MA). Rabbit antibody against NHE1 (Cat #ab67314) and rat antibody against CD8 (Cat #ab22378) were from Abcam Ltd (Cambridge, MA). Mouse antibody against NHE1 (Cat #sc-136239) was from Santa Cruz Biotechology (Dallas, TX). APC-CD11b, BV510-CD45, PE-Cy5 CD8a, APC/Cy7-CD4, PE-FoxP3, PE-PD-1, APC-CD25 and PE-Cy7-CTLA-4 were obtained from Biolegend. APC-IFNγ and eFluor 450-CD16/32 were purchased from eBioscience and PE-Ym1 (clone EPR15263) was from Abcam. The BrdU Cell Proliferation Assay kit (Cat #2752) was from Millipore (Billerica, MA). Proximity ligation assay (PLA) probe with anti-Rabbit PLUS and anti-Mouse MINUS kit (Cat #DUO920002/DUO92004-100RXN) was from Sigma (St. Louis, MO). BCA Protein Assay Kit (Cat #23227) was from Thermo Scientific (Rockford, IL).

**Immunostaining image analysis**: Confocal images of immunofluorescence-stained brain sections (25 μm) were acquired with a Leica DMIRE2 inverted confocal laser scanning microscope, using a 40x objective, identical pinhole, intensity, and exposure parameters were applied for all the images to be compared. Images were computer processed in Image J (National Institutes of Health), Adobe photoshop CC and Adobe illustrator softwares. Background intensity in each original image was subtracted using Image J software as described previously ^1^.

**Cell counting**: In a blinded manner, positively stained cells (Ki67, CD-8, or Caspase-3) were counted in the tumor or in the tumor border of coronal brain sections (n=4) using ImageJ Cell Counter plugin. The positive puncta signals localized to the cytoplasma were counted for the Ki67, CD-8 and Caspase-3 positive immunosignals. For negative controls, brain sections were stained with isotype control antibodies followed by respective secondary antibodies. In each section, 3-4 randomly selected fields were analyzed and averaged per brain section. The number of cells that were positively stained with anti-Ki67, anti-CD-8, or anti-caspase-3 in each area was normalized and expressed as a percentage of immunolabeled cells relative to the total number of cells assessed by TO-PRO-3 in each area.

**Fluorescence intensity quantification:** To quantify NHE1 protein expression, representative images from 3-4 regions per brain section (n=5) were taken under identical camera and microscope settings. For every image, the mean intensity values were measured using Image J software.

**NHE1 full-length blot of SB28 glioma cells for Figure 1C**

**
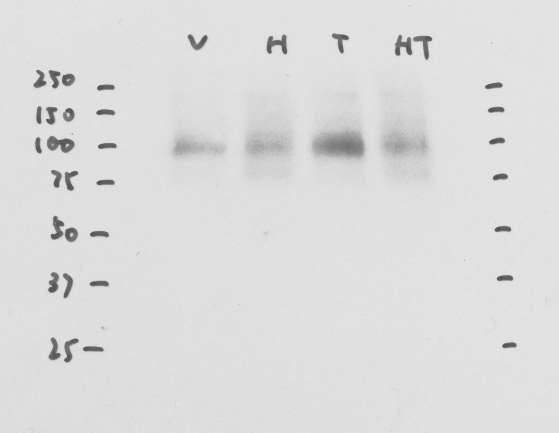
**

**β-actin full-length blot of SB28 glioma cells for Figure 1C**

**
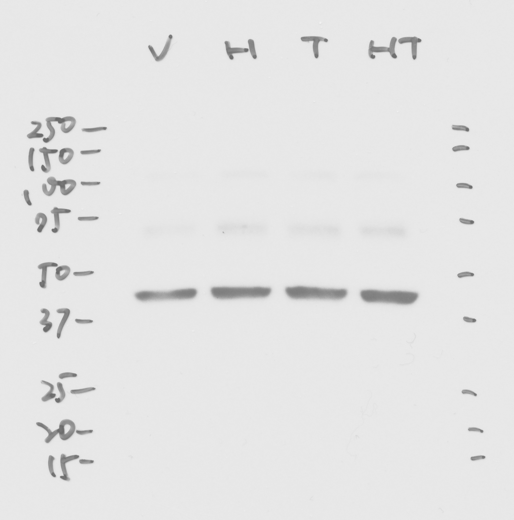
**

**Fig. S1. Western blot supplementary materials for Figure 1C.**


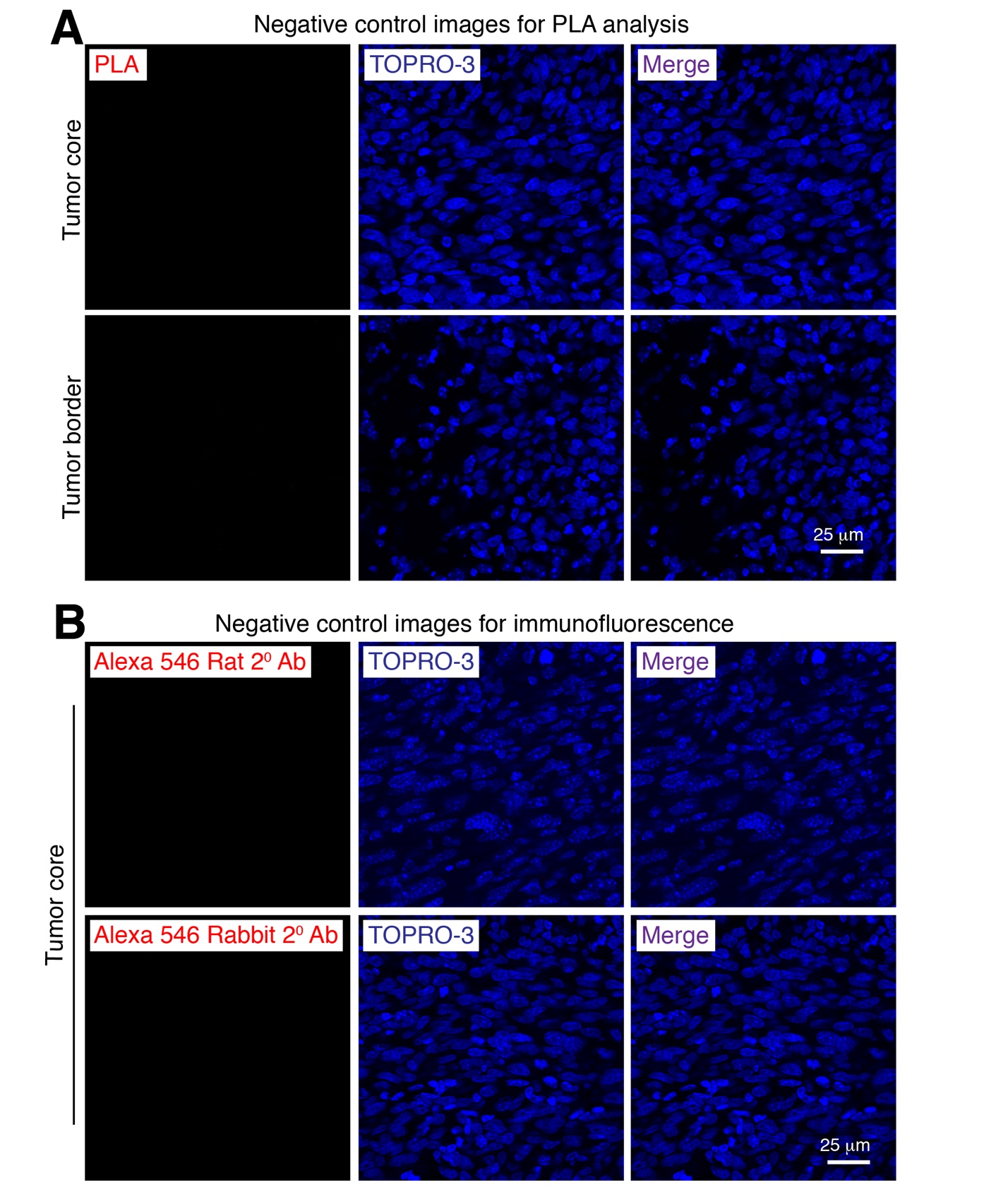


**Fig. S2. Representative negative control images**

**A**. Brain sections were incubated with primary anti-MMP9 antibody alone at 4^o^C overnight followed by incubation with anti-rabbit PLA MINUS and anti-mouse PLA PLUS secondary probes. All other steps were similar to the double-antibody labeling in PLA as described in Methods section.

**B**. Representative images of isotype negative controls for NHE1, Ki67, cleaved caspase-3, and CD8 staining. Brain sections were incubated with isotype anti-rabbit IgG control (NHE1, Ki67, and cleaved caspase-3) or isotype anti-rat IgG2b control (CD8) at 4^o^C overnight followed by incubation with secondary antibody (Alexa 546 rabbit secondary or Alexa 546 rat secondary antibody).

**
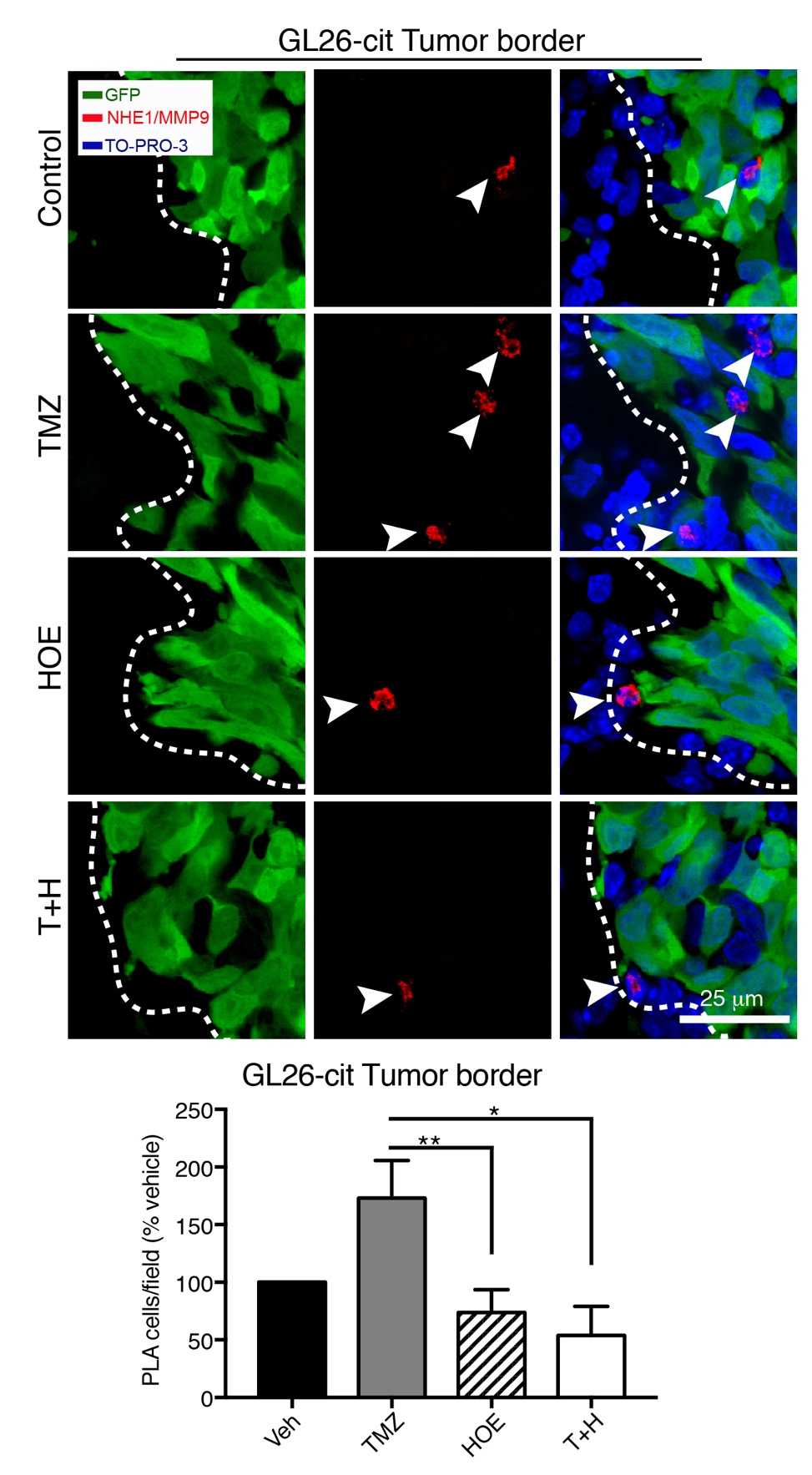
**

**Fig. S3. Proximity ligation assay for NHE1-MMP9 interactions in brain tumor tissues**

Representative confocal images of GFP and PLA immunofluorescence signal (**arrow head**) near the tumor border **(dashed lines)** in mice transplanted with GL26-Cit cells. Summary data of PLA^+^ positive cells were expressed as % of vehicle control. Data are means ± SEM (n=6-7). * p < 0.05; ** p < 0.01.


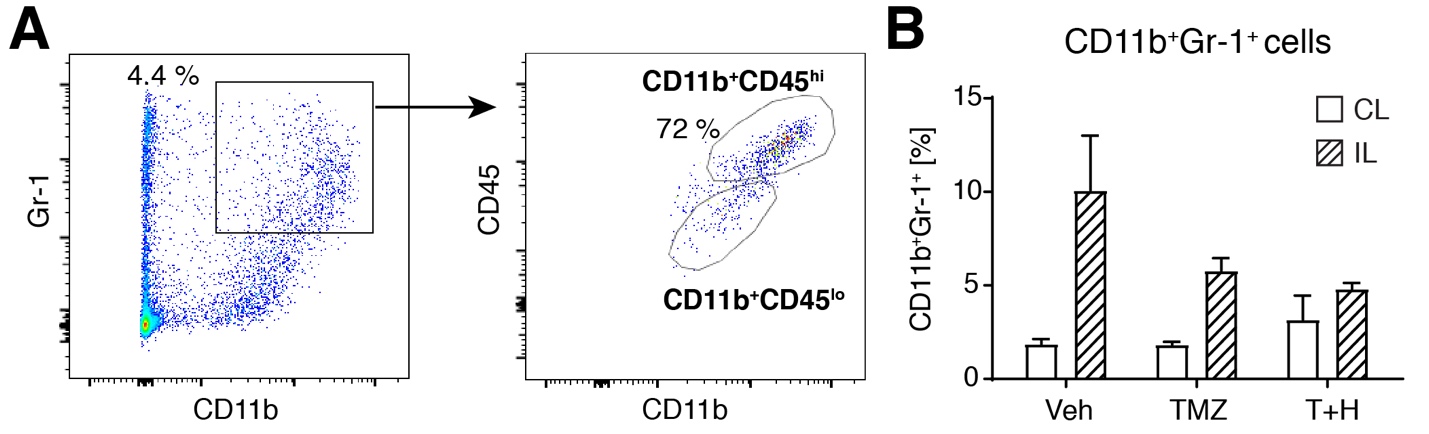


**Fig. S4. Myeloid-derived suppressor cells within CD11b^+^CD45^hi^ population.**

**A**. Representative flow cytometric profile illustrating gating of CD11b^+^/Gr-1^+^ and CD11b^+^/CD45^hi^ population. **B**. Percentage of myeloid derived suppressor cells (CD11b^+^/Gr-1^+^) in non-tumor bearing contralateral (CL) hemispheres and ipsilateral SB28 tumor tissues (IL) from mice treated with Veh (PBS-DMSO), TMZ and T+H (as described in **Fig. 6**). All data are mean ± SEM, n=2.

**
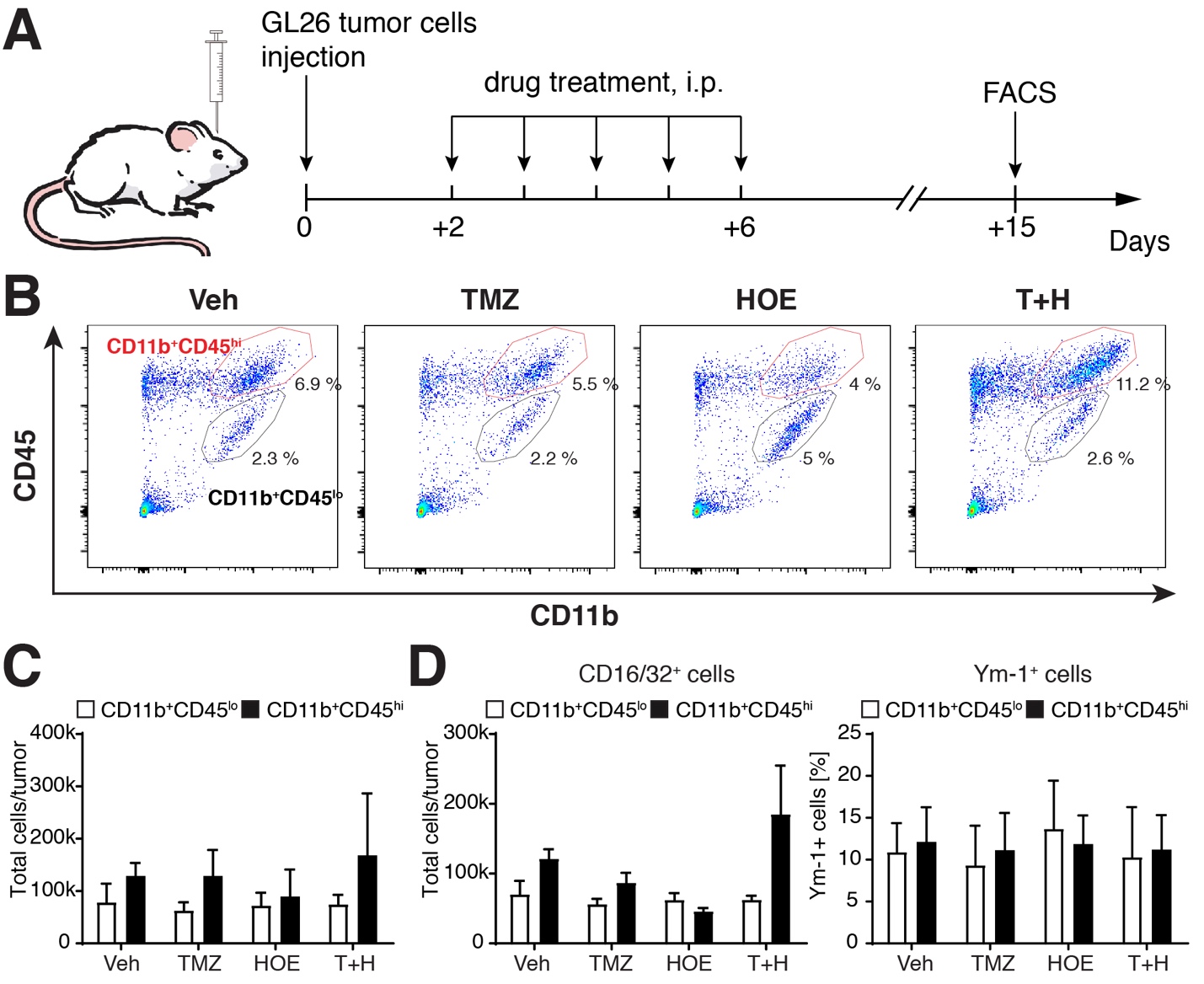
**

**Fig. S5. Effects of NHE1 blockade in combination with TMZ treatment on anti-tumor pro-inflammatory TAMs of GL26 tumors**

**A**. Mice received either Veh (PBS-DMSO), HOE642, TMZ or T+H combination treatments for 5 consecutive days. Mice were sacrificed at 15 d.p.i. and flow cytometric analysis of tumor tissues was performed. **B**. Representative flow cytometric profile showing gating strategy of microglia (CD11b^+^/CD45^lo-med^) and infiltrating myeloid cells (CD11b^+^/CD45^hi^). **C**. Total number of microglia and infiltrating myeloid cells. **D**. Inflammatory profile of CD11b^+^/CD45^lo^ and CD11b^+^/CD45^hi^ cells. All data are mean ± SEM, n=3.


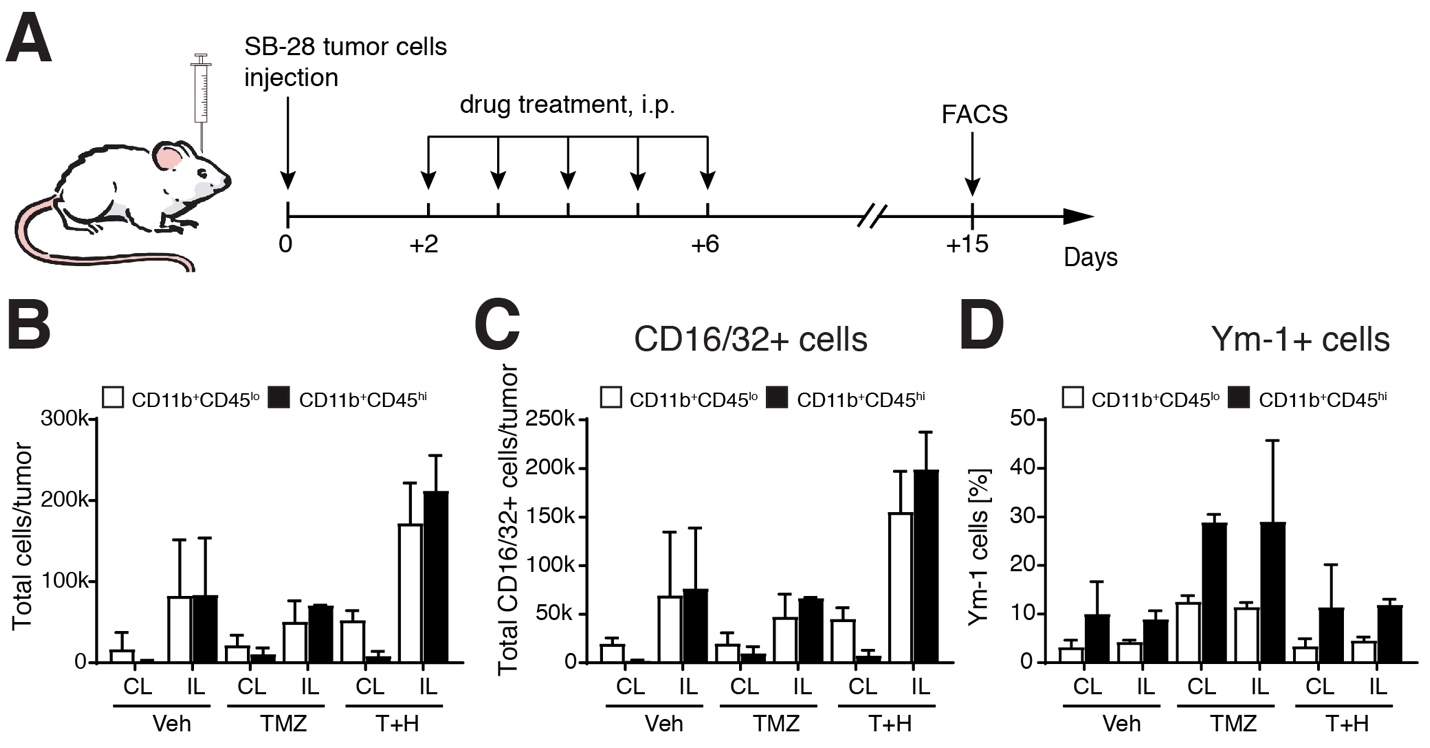


**Fig. S6. Effects of treatments on immune cells from non-tumor brain tissues**

**A**. Mice received either Veh (PBS-DMSO), HOE642, TMZ or T+H combination treatments for 5 consecutive days as described before. Mice were sacrificed at 15 d.p.i. and flow cytometric analysis was performed. **B**. Total number of microglia (CD11b^+^/CD45^lo^) and infiltrating myeloid cells (CD11b^+^/CD45^hi^) in non-tumor contralateral (CL) hemisphere tissues and ipsilateral (IL) tumor tissues. **C, D**. Inflammatory profiles of microglia and infiltrating myeloid cells. All data are mean ± SEM, n=2.

**
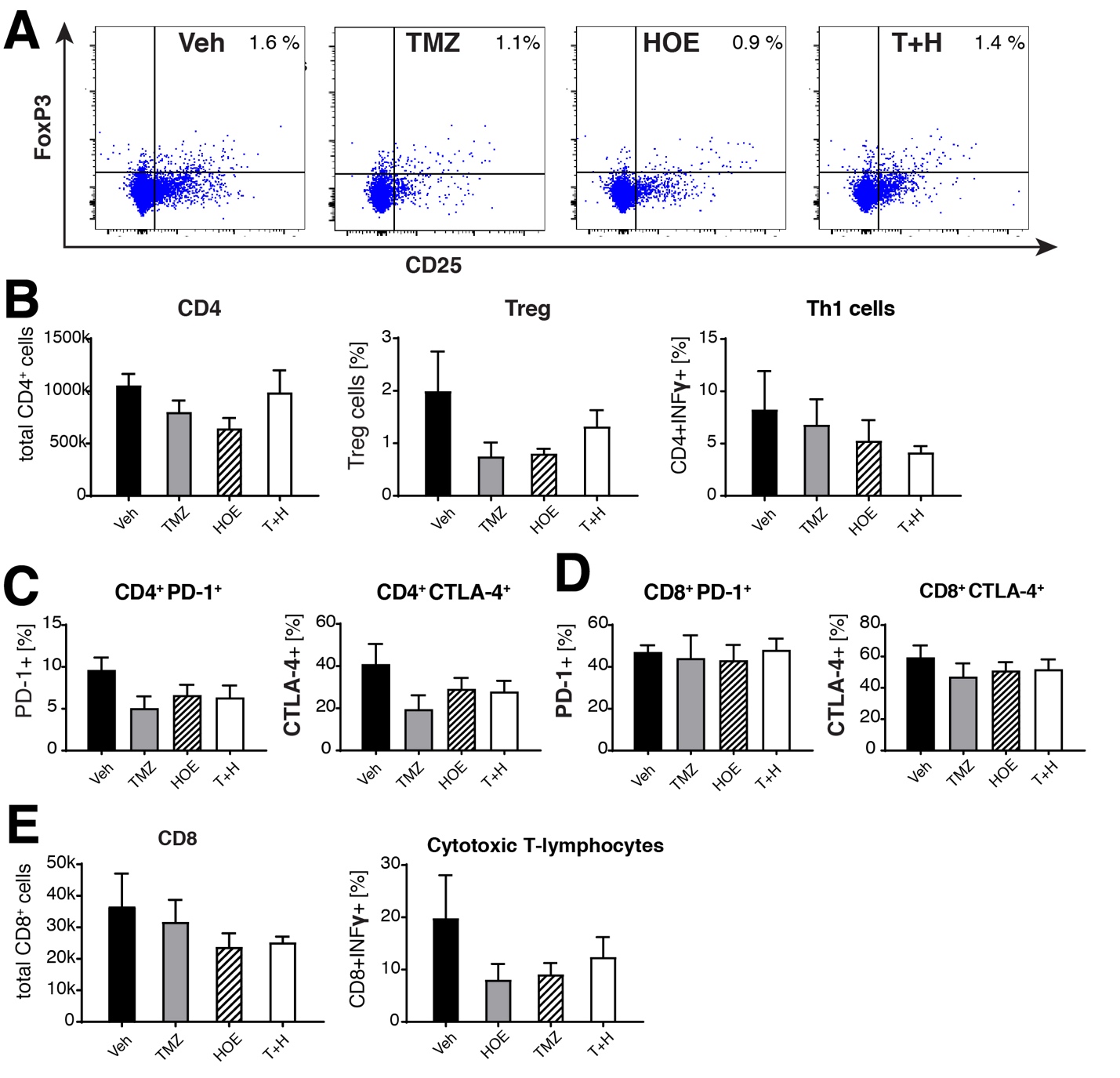
**

**Fig. S7. Flow cytometric profiles of T cells in GL26 glioma**

**A**. Representative flow cytometric profile of CD4^+^CD25^+^FoxP3^+^ (Treg). **B**. Total CD4^+^ T cell counts and percentage of CD4^+^IFNγ^+^ and Treg cells within CD4^+^ population. **C**. Percentage of PD-1 and CTLA4 expression in CD4^+^ T cell population. **D**. Percentage of PD-1 and CTLA4 expression in CD8^+^ T cell population. **E**. Total CD8^+^ T cell counts and percentage of IFNγ^+^ cells in CD8^+^ population. Data are mean ± SEM, n=5-7.


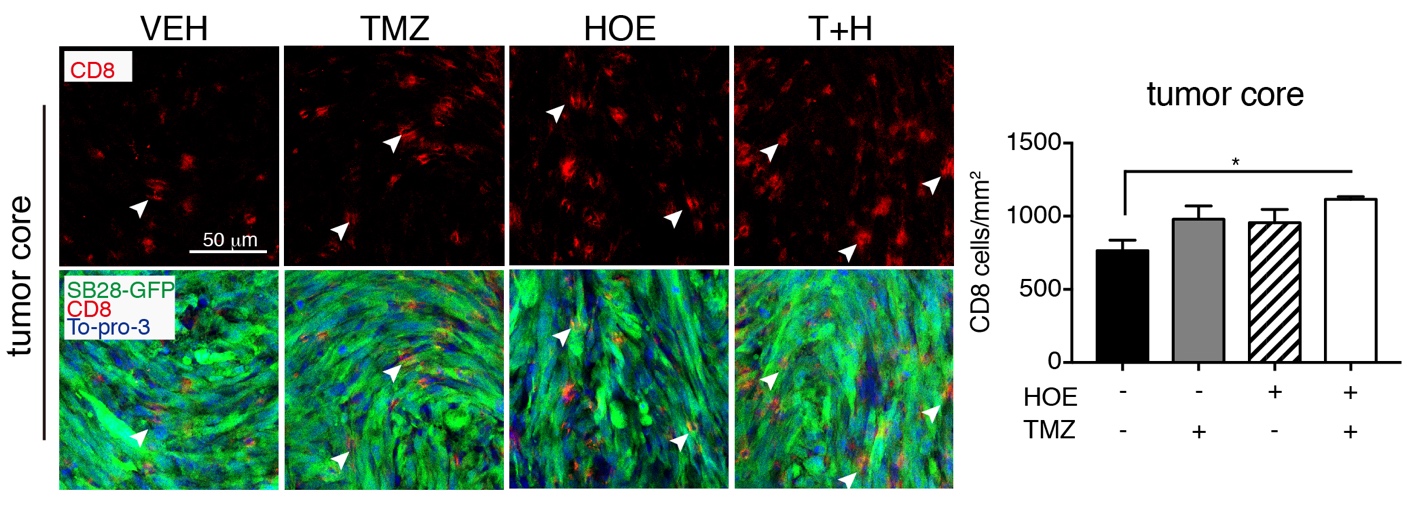


**Fig. S8. Infiltration of CD8^+^ T cell in SB28-GFP tumor core**

Representative immunostaining of fixed brain sections (25 µm thickness) for CD8 protein expression in SB28 tumor core. Data are mean ± SEM (n=5). *p < 0.05.

**
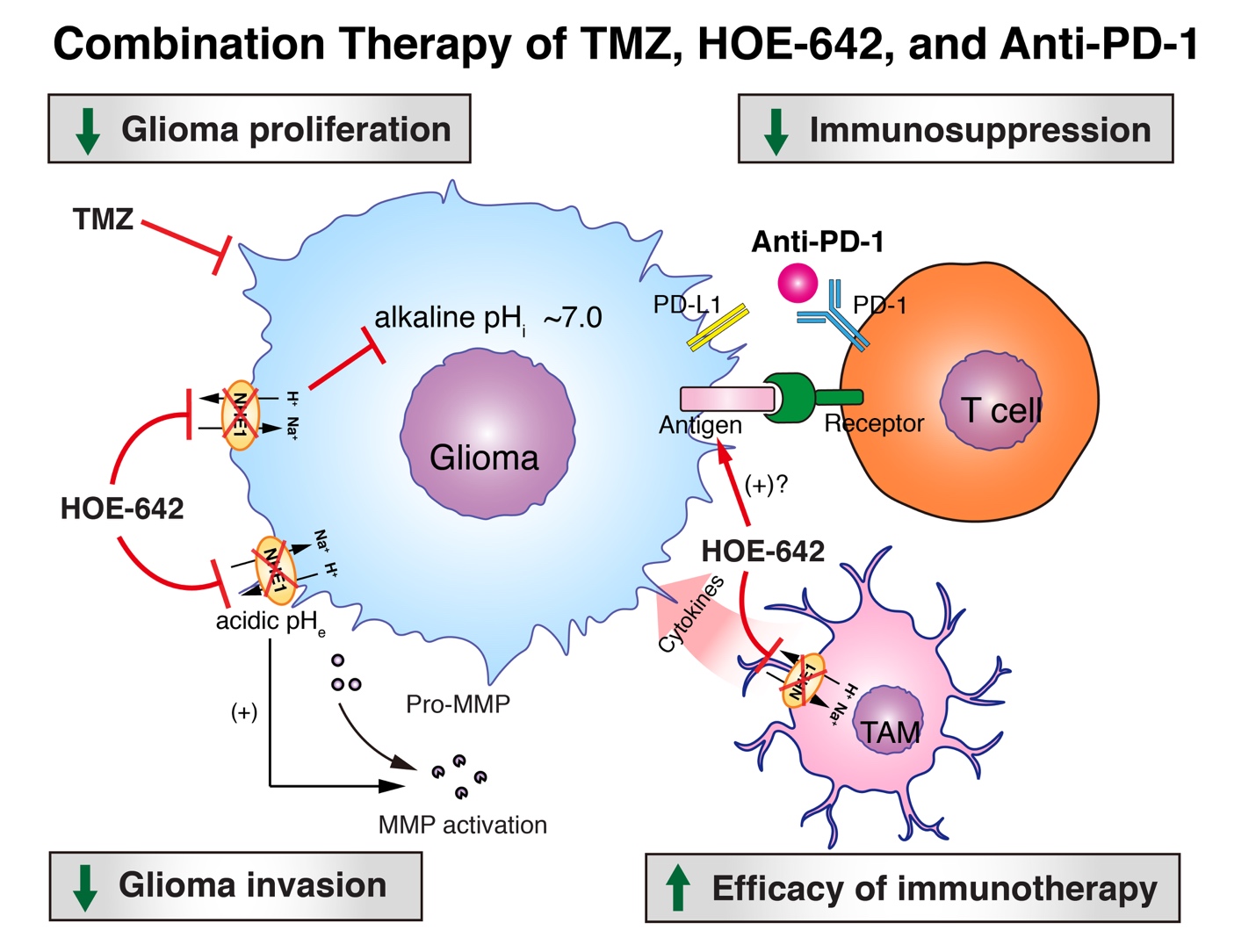
**

**Fig. S9. Blockade of Na/H exchanger stimulates glioma immunogenicity and increases TMZ and anti-PD-1 therapy efficacy.**

Na/H exchanger (NHE1) plays a role in glioma progression via multiple mechanisms. First, NHE1-mediated H^+^ efflux maintains glioma alkaline pH_i_ and cell proliferation. NHE1 activation promotes glioma tumor invasion via maintaining acidic pH_e_, which stimulates metalloprotease (MMP) family proteins. In response to temozolomide (TMZ) treatment, glioma cells increased NHE1 protein expression and developed resistance to TMZ-mediated apoptosis. Administration of NHE1 inhibitor HOE-642 together with TMZ significantly decreased glioma proliferation, and increased glioma apoptosis and animal overall survival. In addition, blockade of NHE1 stimulated pro-inflammatory polarization of tumor-associated microglia and macrophages (TAMs) and increased infiltration of T cells into tumor tissues. Importantly, combination therapy of TMZ, NHE1 inhibitor, and anti-PD-1 antibody significantly extended median survival of the glioma-bearing mice. Therefore, blocking NHE1 protein is a novel strategy to improve combinatorial TMZ chemotherapy and immunotherapy for glioma.

**References**

1 Begum, G. *et al.* Inhibition of WNK3 Kinase Signaling Reduces Brain Damage and Accelerates Neurological Recovery After Stroke. *Stroke* **46**, 1956-1965, doi:10.1161/STROKEAHA.115.008939 (2015).
